# Estimation of community-level influenza-associated illness in a low resource rural setting in India

**DOI:** 10.1371/journal.pone.0196495

**Published:** 2018-04-26

**Authors:** Siddhartha Saha, Vivek Gupta, Fatimah S. Dawood, Shobha Broor, Kathryn E. Lafond, Mandeep S. Chadha, Sanjay K. Rai, Anand Krishnan

**Affiliations:** 1 Influenza Program, US Center for Disease Control and Prevention-India office, New Delhi, India; 2 Centre for Community Medicine, All India Institute of Medical Sciences, New Delhi, India; 3 Influenza Division, US Centers for Disease Control and Prevention, Atlanta, Georgia, United States of America; 4 Department of Microbiology, All India Institute of Medical Sciences, New Delhi, India; 5 National Institute of Virology, Pune, India; University of Washington, UNITED STATES

## Abstract

**Objective:**

To estimate rates of community-level influenza-like-illness (ILI) and influenza-associated ILI in rural north India.

**Methods:**

During 2011, we conducted household-based healthcare utilization surveys (HUS) for any acute medical illness (AMI) in preceding 14days among residents of 28villages of Ballabgarh, in north India. Concurrently, we conducted clinic-based surveillance (CBS) in the area for AMI episodes with illness onset ≤3days and collected nasal and throat swabs for influenza virus testing using real-time polymerase chain reaction. Retrospectively, we applied ILI case definition (measured/reported fever and cough) to HUS and CBS data. We attributed 14days of risk-time per person surveyed in HUS and estimated community ILI rate by dividing the number of ILI cases in HUS by total risk-time. We used CBS data on influenza positivity and applied it to HUS-based community ILI rates by age, month, and clinic type, to estimate the community influenza-associated ILI rates.

**Findings:**

The HUS of 69,369 residents during the year generated risk-time of 3945 person-years (p-y) and identified 150 (5%, 95%CI: 4–6) ILI episodes (38 ILI episodes/1,000 p-y; 95% CI 32–44). Among 1,372 ILI cases enrolled from clinics, 126 (9%; 95% CI 8–11) had laboratory-confirmed influenza (A (H3N2) = 72; B = 54). After adjusting for age, month, and clinic type, overall influenza-associated ILI rate was 4.8/1,000 p-y; rates were highest among children <5 years (13; 95% CI: 4–29) and persons≥60 years (11; 95%CI: 2–30).

**Conclusion:**

We present a novel way to use HUS and CBS data to generate estimates of community burden of influenza. Although the confidence intervals overlapped considerably, higher point estimates for burden among young children and older adults shows the utility for exploring the value of influenza vaccination among target groups.

## Introduction

Influenza is recognized to be an important cause of morbidity and mortality worldwide including low- and middle-income countries (LMICs) in tropical regions but data on influenza disease burden in most of these countries remain sparse [1]. In addition, most burden studies focus on influenza-associated hospitalization [2], even though outpatient and non-medically attended influenza infections have been recognized as important considerations when estimating public health impact and cost [3]. Further, the contribution of non-medically attended illness to total influenza disease burden likely varies across settings and has not been thoroughly studied in LMICs. Data on burden estimates and identification of risk groups are crucial for prioritizing influenza prevention and control measures especially in low resource settings of LMICs. Currently, the Government of India recommends influenza vaccination for health care workers, pregnant women and persons with chronic diseases with vaccine being desirable for elderly (> = 65 years) and children (6 months-8years age).[4]

One reason for the lack of data on outpatient and non-medically attended illness is that community-based burden studies with laboratory confirmation of influenza virus infection are resource-intensive and challenging to conduct. In some high-income countries where the catchment populations that attend health facilities are known, the incidence of outpatient influenza-like illness (ILI) and influenza-associated ILI [5, 6] have been estimated. However, in LMICs, such as China and Bangladesh, estimates of influenza-associated ILI mainly focused on children <5 years old or presented disaggregated rates for children over 5 years [7, 8]. In India, estimating outpatient and non-medically attended illness is particularly challenging due to the large number and diversity of private clinics which are challenging to enumerate, and vast health care seeking within this large outpatient private sector; and the lack of defined catchment populations in many areas. In addition, the ILI surveillance from around the study area showed that unlike temperate regions, influenza circulates round the year and peaks during monsoons (July-September) with a smaller peak in March during some years[9]. Therefore, the seasonality of influenza also need to be considered while estimating burden. The World Health Organization (WHO) provides guidelines on using clinic-based ILI surveillance data for burden estimations in the absence of a well-defined catchment population [10]. We used a similar approach to estimate community-level rates of total ILI and influenza-associated ILI, including outpatient and non-medically attended episodes, in a rural Health and Demographic Surveillance System (HDSS) site in Ballabgarh, Haryana (north India).

## Methods

### Study site

Ballabgarh is a block of Faridabad district in the state of Haryana which is located approximately 40 km south of New Delhi, India. The HDSS site at Ballabgarh has been previously described [11]. Briefly, the HDSS site includes 28 villages with a total population of approximately 90,240 in 2011 as per the local annual census. The HDSS database is kept up-to-date through a monthly vital event registration process. Public health facilities in the HDSS include two primary health centers (PHCs) that provide basic health care in their outpatient departments (OPD) and one 50-bed secondary level facility providing in-patient care. In addition, weekly outreach clinics are conducted at 10 health sub-centers. The community also has access to numerous private health facilities that provide inpatient and outpatient health services. The majority of the private clinics with qualified medical professionals are located in Ballabgarh and Faridabad towns and provide inpatient (5–200 beds) as well as outpatient services. In addition, there are small clinics within the HDSS villages which are mostly run by unqualified medical practitioners and commonly visited by the community for minor ailments.

### Healthcare utilization survey (HUS)

From July 2010 to January 2012, community-based cross-sectional healthcare utilization surveys (HUS) were conducted across the study villages. Trained field investigators visited an average of 365 households per week and interviewed at least one adult respondent in each visited household. All household members registered with the HDSS system were eligible for inclusion. Information on whether residents in the household had experienced any acute medical illness (AMI) during the preceding 14 days was collected We defined AMI as any illness irrespective of symptoms excluding injury and those related to obstetric or surgical problems with onset in the past 14 days among any member of the household. We collected detailed symptom data for each AMI episode using a structured interview (S1 File). We also collected data on type of facility (public vs. private) and health-care (outpatient vs. inpatient) sought for medically attended illness. As we were only measuring incidence of influenza associated illness not requiring hospitalization, AMI cases with history of inpatient admissions for the current episode were excluded.

### Clinic based surveillance (CBS)

During December 2010 to January 2012, systematic surveillance was conducted in the outpatient clinics of PHCs and outreach clinics at sub-centers to enroll AMI patients defined similarly to that used for the HUS but with illness onset within the preceding three days; AMI cases with onset more than 3 days were excluded to increase the probability of virus detection. We also identified key private practitioners in the study villages who were identified on the HUS as frequently used for medical care by community members and systematically enrolled patients with AMI from these private clinics. The study staff systematically visited two to three outpatient clinics and enrolled the first five eligible cases per clinic with an average of 10–12 AMI cases per day, collecting data from each case on clinical symptoms and signs using structured interviews and a brief clinical exam.

### Specimen collection and testing

Nasal and throat swabs were collected only from AMI patients enrolled at clinics, placed together in viral transport media, immediately stored in cold boxes or refrigerated, and transported to the laboratory within 48 hours of collection for further processing of samples. All specimens were tested at the All India Institute of Medical Sciences (New Delhi, India) using real-time reverse transcription polymerase chain reaction (rRT-PCR) for influenza A (A(H1N1), A(H1N1pdm09), and A(H3N2) and B viruses using CDC protocols [12]. Samples positive for influenza by rRT-PCR were inoculated in Madine Darby Canine Kidney cells for virus isolation followed by haemagglutination inhibition for influenza virus identification and subtyping.

### Data analysis

**Fig 1:**
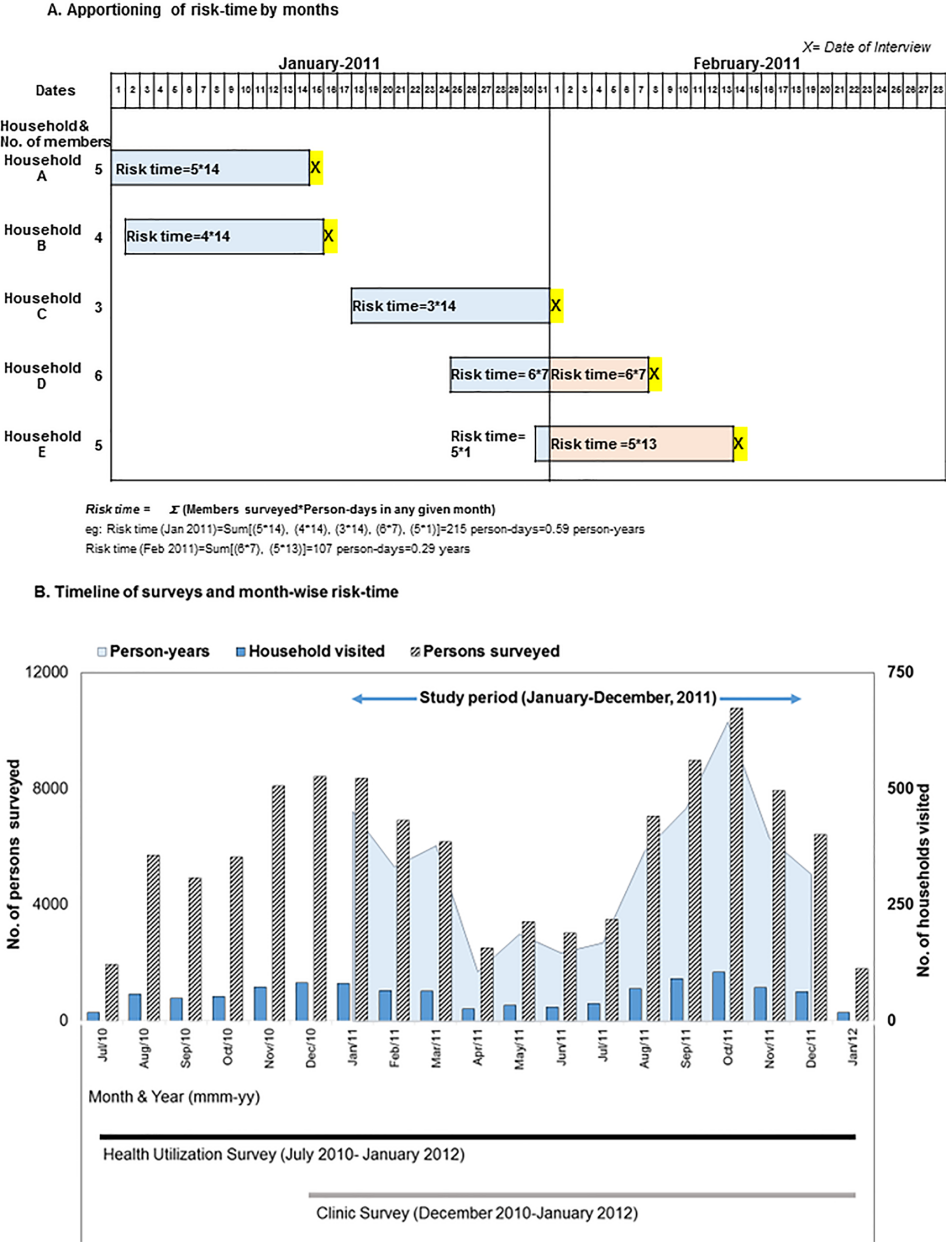
Schematic diagram depicting estimation of person risk-time. **A.** shows estimation of risk-time and apportioning by month. For each surveyed household, all the members contributed risk-time of 14 days preceding the date of interview. Risk time for any month was estimated by summation of person-days of risk-time contributed for that month by members of each household surveyed. **B.** shows timeline of surveys and month-wise risk time in person-years. The solid columns indicate number of households visited and striped columns indicate number of persons surveyed. The shaded area depicts the risk-time in person-years contributed by persons surveyed and apportioned by months.

**Fig 2:**
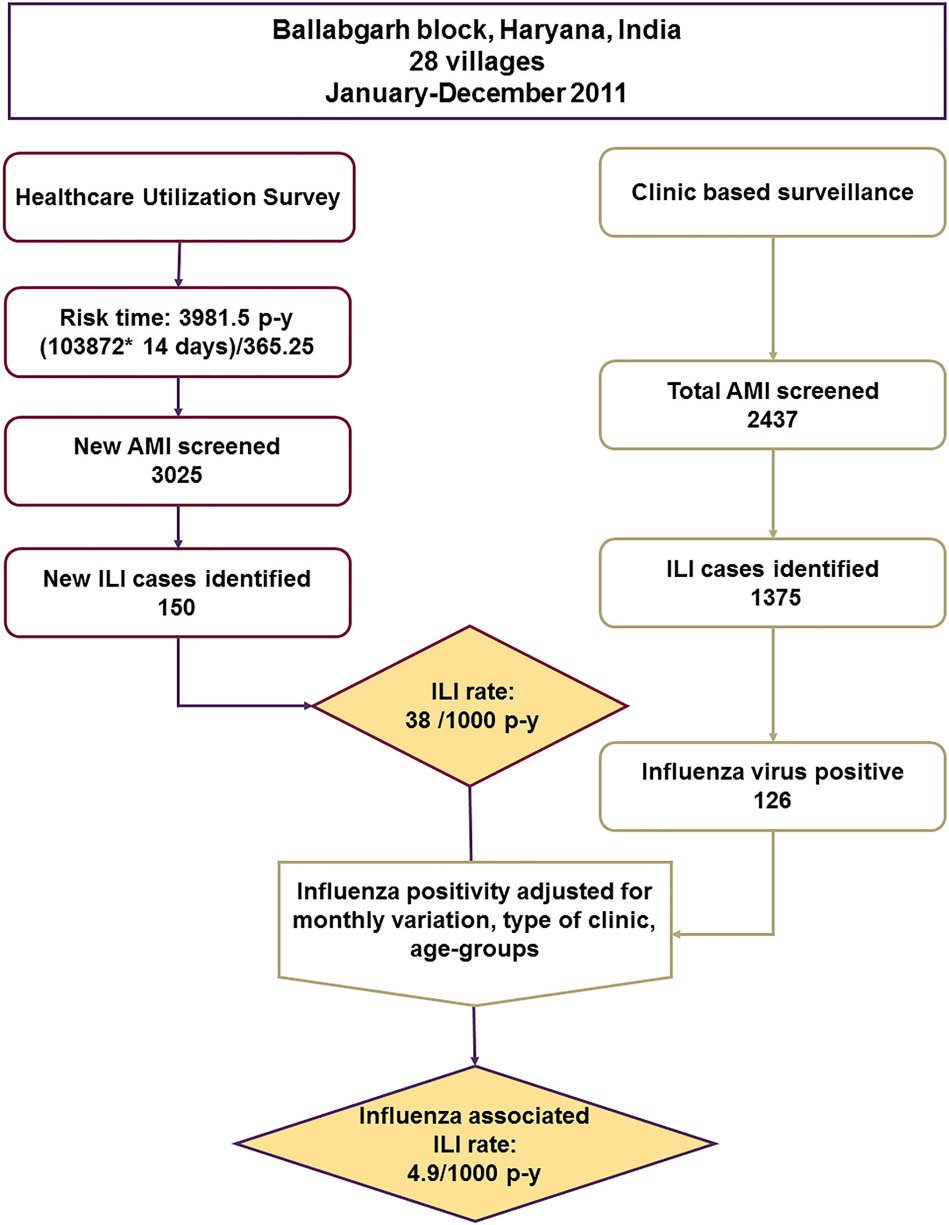
Flow-chart depicting study methods for estimation of rates of influenza-associated ILI* in 28 villages in the Ballabgarh block (Haryana, India)–January 1 to December 31, 2011. (*ILI: influenza like illness defined as cough with history/measured fever. **AMI: acute medical illness defined any illness irrespective of symptoms excluding injury and those related to obstetric or surgical problems. ***p-y: person-years).

For the current study, we used concurrently collected data from the HUS and CBS from January to December 2011 (Figs 1 and 2). We filtered only ILI cases defined as presence of measured/reported fever and cough from the HUS and CBS data, which is similar to the WHO case definition with the exception of the duration of onset [10]. We attributed 14 person-days of risk-time for each individual in the household surveyed during the HUS and then apportioned the risk days into corresponding calendar months to convert into risk-months (Fig 1). We then calculated monthly ILI rates by dividing the number of ILI cases identified during the 14 day HUS risk period by the cumulative risk-time calculated for each calendar month for each age-group. We used the laboratory-confirmed influenza clinic data to estimate the proportion positive with influenza virus during each month among AMI cases enrolled in outpatient clinics who met the ILI case definition.

Assuming similar prevalence of influenza virus infection among ILI cases in both clinics and households, we estimated the influenza-associated ILI cases in the community by multiplying the proportion of ILI cases positive for influenza in the clinics by ILI cases in the community identified on the HUS; we performed these calculations by month, age group and type of health facility (i.e., public versus private). We then estimated age-stratified and total incidence of influenza-associated ILI in the community by dividing the estimated number of influenza-associated ILI cases in the community by person-year of risk time. We used chi-square to test for statistical significance between the proportions. We calculated 95% confidence intervals (95% CI) for the rates assuming a Poisson distribution. We used Stata/SE 14.1 (StataCorp LP) and MS Excel 2013 for data analysis.

### Ethical clearance

The study protocol was approved by the institutional ethics committees of the All India Institute of Medical Sciences, New Delhi; National Institute of Virology, Pune in India, and U.S. Centers for Disease Control and Prevention, USA. We requested written informed consent from all patients enrolled in outpatient clinics and heads of households included in the HUS.

## Results

During the study period, 69,369 individuals in 11,816 households were reached at least once with a total of 103,872 contacts (33,217 persons were surveyed twice and 1,286 were surveyed three times) during the study period. Assuming 14 days of risk-time for each contact, the total cumulative risk-time for all age groups was 3945 person-years (p-y) (Table 1). From the HUS, 3,025 AMI cases were identified in the community, of which 150 (5.0%, 95%CI: 4.2–5.8) met the ILI case definition, resulting in an ILI rate of 38 episodes/1000 p-y (95% CI 32–44 episodes/1000p-y). The ILI rates were among children aged <5 years (108.3, 95%CI: 79.5–143.1) was significantly higher than other age groups (p<0.01). Approximately half (47%) of ILI cases sought treatment from clinics (private sector: 34.7%; public sector: 12.0%); the rest sought treatment from local unqualified practitioners (51.3%), pharmacists (0.7%), or did not seek care at all (1.3%). There was no significant difference in preference by age-groups for seeking care in any particular type of clinic.

**Table 1:** Health utilization survey (HUS) data on the frequency influenza-like illness (ILI)* in a rural community, by age, Ballabgarh, India, 2011.

Age-groups	Person-years of risk time	Persons with acute medical illness (AMI) detected in community	Persons with influenza like illness (ILI) detected in community	Proportion of AMI cases that met ILI case definition
	(%)	n (%)	n (%)	% (95%CI)**
0–4y	397.3 (10.1)	555 (18.3)	43 (28.7)	7.7 (5.7–10.3)
5–14y	824.3 (20.9)	690 (22.8)	24 (16.0)	3.5 (2.2–5.1)
15–59y	2443.7 (61.9)	1517 (50.1)	72 (48.0)	4.7 (3.7–5.9)
> = 60y	280 (7.1)	263 (8.7)	11 (7.3)	4.2 (2.1–7.4)
All ages	3945.2(100)	3025 (100)	150 (100)	5.0 (4.2–5.8)

* Influenza-like-illness was defined as cough and reported or presence of fever.

** Proportion of ILI cases is significantly different between age groups (p<0.01)

During the same period, 2,431 AMI (private sector 2030; public sector 401) cases were screened and 1,372 ILI cases (56.4%, 95%CI: 54.4–58.4) were detected in the CBS (Table 2). The majority of ILI cases were from public facilities (1141, 83.2%). The age group and gender distribution of AMI and ILI cases enrolled in the clinics was similar to the AMI and ILI cases detected in the community; the seasonal distribution of ILI in the community and in clinics was also similar (data not shown).

**Table 2:** Distribution of influenza-like illness (ILI) and influenza associated ILI in enrolled clinics, by age, Ballabgarh, India, 2011.

Age-groups	No. of patients with acute medical illness(AMI) screened in clinics	No. of influenza like illness cases in clinics	Proportion of AMI cases in clinics that met ILI case definition %	No. of patients with laboratory-confirmed influenza in clinics	Proportion of ILI cases with laboratory-confirmed influenza %
	n (%)	n (%)	(95%CI)*	n (%)	(95%CI)**
0–4y	325 (13.4)	211 (15.4)	64.9 (59.5–70.1)	16 (12.7)	7.6 (4.4–12.0)
5–14y	613 (25.2)	358 (26.1)	58.4 (54.4–62.3)	46 (36.5)	12.8 (9.5–16.8)
15–59y	1119 (46.0)	626 (45.6)	55.9 (53.0–58.9)	56 (44.4)	8.9 (6.8–11.5)
> = 60y	374 (15.4)	177 (12.9)	47.3 (42.2–52.5)	8 (6.3)	4.5 (2.0–8.7)
All ages	2431 (100)	1372 (100)	56.4 (54.4–58.4)	126 (100)	9.2 (7.7–10.8)

* Proportion of ILI cases is significantly different between age groups (p<0.01).

**Influenza positivity is significantly different between age groups (p = 0.01)

Of the ILI cases identified through clinic surveillance, 126 (9.2%, 95%CI: 7.7–10.8) tested positive for influenza viruses, of which 72 (57%) were positive for influenza A(H3N2) and 54 (43%) for influenza B virus. The prevalence of laboratory-confirmed influenza virus among ILI patients attending clinics was significantly different (p = 0.01) between age groups with highest prevalence among the 5–14 year olds (12.8%, 95% CI: 9.5–16.8). We also tested non-ILI AMI cases enrolled in the clinics, and found influenza positivity among non-ILI clinic attendees (26/1059; 2.5%; 95%CI: 1.6–3.6) was lower than the ILI cases. The monthly prevalence of laboratory-confirmed influenza infection among all ILI cases ranged from 0–25% with the highest prevalence occurring during July through September.

**Fig 3:**
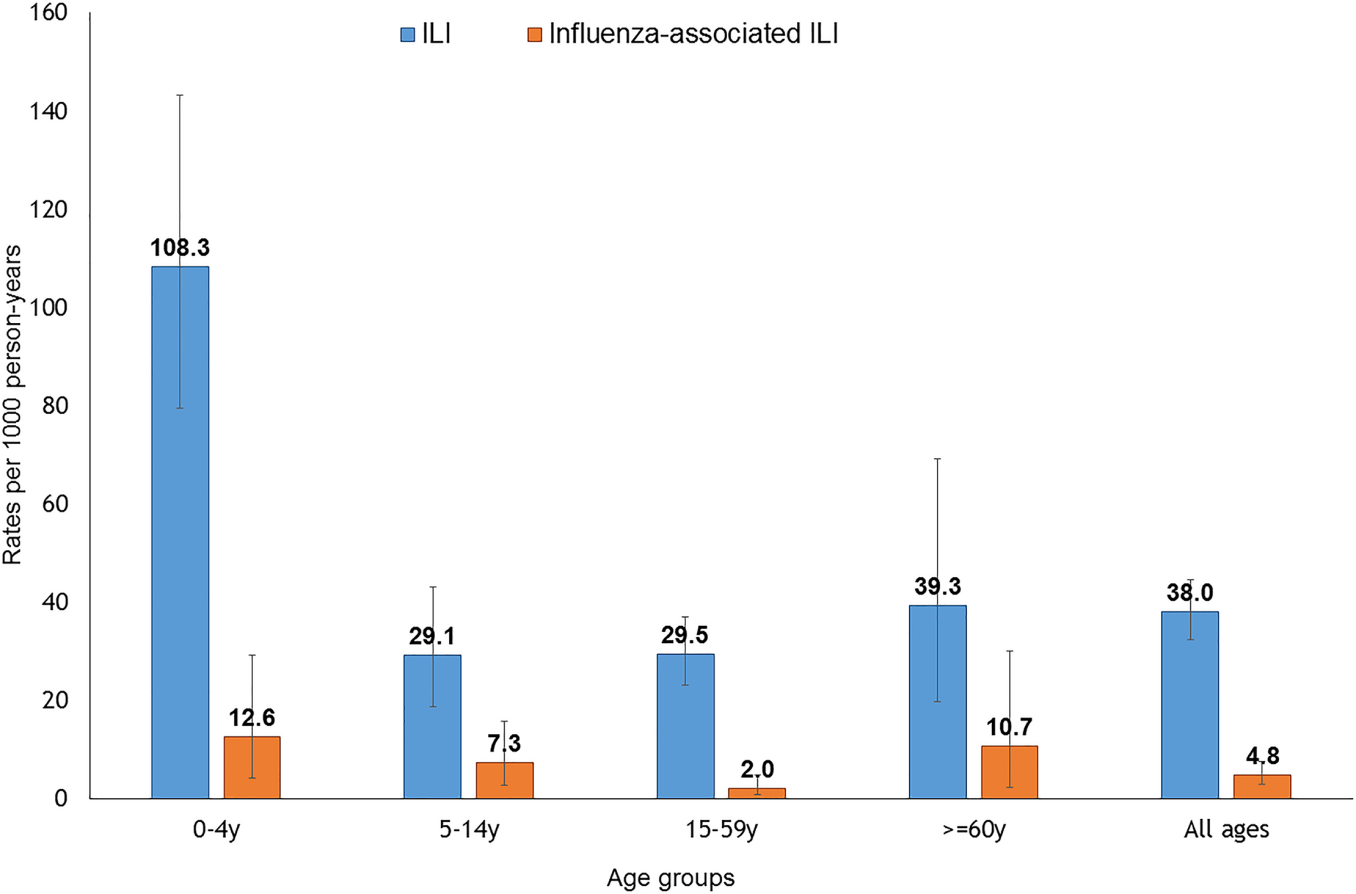
Rates and 95% confidence intervals per 1,000 person-years of influenza-like illness (ILI) and influenza-associated ILI by age, Ballabgarh, India, 2011. (Vertical axis denotes rates per 1000 person-years and horizontal axis denotes age-groups. The ILI rates are shown in blue striped columns while influenza-associated ILI is shown in purple solid columns. Whiskers indicate 95% confidence intervals).

Overall, the estimated influenza-associated ILI rate during 2011 was 4.8 (95% CI: 2.9–7.5) per 1000 p-y (Fig 3). Influenza-associated ILI rates were highest among children <5 years (12.6; 95% CI: 4.1–29.1), followed by older adults aged ≥60 years (10.7; 95% CI 2.2–30.1), although the differences in age-specific rates were not statistically significant.

## Discussion

Using a novel method using existing HUS, clinic, and virological data, we demonstrate that young children and older adults experienced the highest rates of both community ILI and influenza-associated ILI during 2011 in our study population in a rural northern Indian village. This is consistent with age trends in other LMICs with similar data, including Bangladesh, Brazil, and China [7, 8, 13], as well as other higher income countries [14]. The WHO guideline for influenza associated disease burden estimation suggests health facility records to define the catchment area and determine the denominator for estimation of rates. However, we opted for modified strategy and used community based survey which revealed that less than half of ILI cases in the community sought care from qualified practitioners and a large proportion of those sought care from private clinics. This modified approach enabled us to estimate the rates of influenza associated with ILI in the community as well. Although not statistically significant, the higher rates among children and older adults in our study is consistent with data from other studies showing the need for development of targeted influenza vaccination strategies. This data should be able to contribute to the evidence for appropriate vaccine policy recommendations for children and elderly in India.[4] This should also guide clinicians in appropriate case management and use of antivirals in patients with suspected influenza in the absence of laboratory confirmation of influenza, which often is the case in such low resource settings.

Our estimates of 4.8 (2.9–7.5) per 1000 p-y of influenza-associated ILI were lower than estimates of influenza-associated outpatient visits reported from neighboring countries of Bangladesh (66–170 per 1000 p-y) and Thailand (14.2/1000 p-y) [8, 15]. However, data from these other studies are from different years of surveillance and our estimates are based on a community HUS of ILI, while the other studies are clinic-based. The methodology used in this study is different from the Bangladesh study in terms of case-definition, health care utilization surveys and extrapolation methods. Our lower influenza-associated ILI rates could also be due to lower circulation of influenza virus in our study area during 2011 as sentinel surveillance data also showed lower influenza circulation from the adjacent Delhi area during the same period [9]. A study during 2010–12 also showed lower rates of influenza associated hospitalization during 2011 compared to other years in Vadu HDSS site situated in western India [2]. Similarly, a lower incidence of ILI was observed in United States and Europe during the year compared to other years [14, 16].

Our study also documented that 99% of community residents with ILI sought some form of healthcare for their illness and thus likely incurred some expense associated with their illness. In addition, a large proportion of persons with ILI sought care from providers in the private sector and from local unqualified practitioners, which is common practice in India [17] and has also been observed in other countries [18, 19]. These findings highlight the importance of capturing visits to private sector providers and unqualified practitioners in studies of disease burden in India and other similar LMICs, particularly when estimating public health impact and cost of illness. In addition, public health interventions, including vaccination, that involve medical care providers should consider strategies for including private sector providers and unqualified practitioners to reach a larger proportion of the population in these settings.

Our study has several limitations. First, our approach of relying on a single household adult respondent to provide data for all household members might have led to underreporting of ILI cases, especially those with mild symptoms, leading to an underestimation of ILI and influenza-associated ILI rates in the community. However, we believe that keeping the recall period to the last 14 days likely helped minimize recall issues. Further, given the observation that the majority of community ILI cases had sought clinic-based care, under-estimation due to recall bias is likely to be minimal. Second, although influenza tends to be more severe often requiring medical care, however, we assumed the prevalence of influenza virus infection among the clinic attendees was similar to that in the community, which is difficult to confirm in the absence of laboratory-confirmation among ILI cases in the community. However, similarities in the age and seasonal distribution of ILI cases identified by the HUS and through CBS as well as frequent clinic-based treatment seeking for ILI observed in the HUS support this assumption. Third, we only collected data for a single year although rates of ILI are known to vary substantially across years depending upon circulating influenza virus strains. Further, the confidence interval calculated assuming Poisson distribution around the incidence estimates may not have adequately captured the uncertainties with the sampling system used. Despite these limitations, we demonstrate the use of a community-based estimation approach in a low-income setting to estimate rates of ILI and influenza-associated ILI including both medically-attended and non-medically attended illness. Our study is unique in that it includes both non-medically attended illnesses and illnesses for which care was sought from unqualified providers or the private sector, both of which are often missed in studies of influenza disease burden. Our approach also demonstrates the feasibility of utilizing HDSS platforms for conducting community-based disease burden estimations through a combination of outpatient surveillance and HUS in developing countries where the health facilities frequently lack a defined catchment population.

## Conclusion

We demonstrate the use of an approach similar to the WHO recommended approach for influenza disease burden estimation in a low resource setting. Our study documents that point estimates of rates of ILI and influenza-associated ILI were highest among young children and the older adults in this rural community in northern India in 2011. These findings suggest further studies exploring utility for the development of influenza vaccination strategies for target age groups especially in low and middle income countries.

## Supporting information

S1 File The questionnaires used for community based health utilization survey, interview of persons with acute medical illness in the community, and interview of persons with acute medical illness in the participating health facilities. (PDF)

S2 File De-identified community surveillance file with data on risk time. (CSV)

S3 File De-identified community based survey data of acute medical illness. (CSV)

S4 File De-identified clinic based surveillance data of acute medical illness. (CSV)

S5 File Excel sheet with analytical model used to derive the community based rates for influenza associated ILI. (XLSX)
