# Supplementary material for: Estimation of community-level influenza-associated illness in a low resource rural setting in India
Source: PLoS One. 2018 Apr 26;13(4):e0196495. doi: 10.1371/journal.pone.0196495 (PMC5919664; doi:10.1371/journal.pone.0196495)
Supplement: S1 File — (PDF) [file pone.0196495.s001.pdf]

FAMILY: ___/____(New family number / Total number of new families) HOUSEHOLD HDSS ID: [1] PROJECT [ ] PHC No. [ ][ ] VILLAGE No. [ ][ ][ ][ ] House No.

Influenza Disease Burden in India

Proforma 2: Household Demographic Survey

Date Form Completed: ___ ___ / ___ ___ / ___ ___ ___ ___ (dd/mm/yyyy)

Time of starting Interview		Time of Finishing Interview	
Data Collected By:			

Basic Household Information - ey ?kjsyw lwpuk

**1.** Address – house Number:<br>पता – घर का नम्बर	
**2.** Address - colony name:<br>पता – कालोनी का नाम	
**3.** Phone Number:<br>टेलीफोन नम्बर	

4. Village of residence: निवास का गांव

☐ Ahmedpur (2-64)	☐ Fatehpur Billoch (1-40)	☐ Naryala (2-60)
☐ Atali (2-51)	☐ Garkhera (2-50)	☐ Nawada (1-31)
☐ Behbalpur (1-14)	☐ Jaya (2-61)	☐ Nirhawali (2-63)
☐ Bhatpura (1-43)	☐ Junehra (1-13)	☐ Panehra Kalan (2-62)
☐ Bukharpur (1-20)	☐ Khera (1-12)	☐ Phapunda (1-15)
☐ Chandawli (1-30)	☐ Ladholi (1-41)	☐ Shahpur Kalan (1-42)
☐ Chhainsa (2-70)	☐ Machgar (1-21)	☐ Sahupura (1-34)
☐ Dayalpur (1-11)	☐ Malerna (1-33)	☐ Sotai (1-22)
☐ Dayalpur Colony (1-10)	☐ Maujpur (2-52)	☐ Other (specify):
☐ Duleypur (2-71)	☐ Mujedhi (1-32)	

Family Demographics and Risk Factors
ifjokj tulkf[;dh vkj tkf[ke dkjd

**5.** Total number of persons living in the household:<br>घर में रहने वाले व्यक्तियों की कुल संख्या	___ ___ persons		
**6.** Number of children under the age of 5 in the household:<br>घर में 5 वर्ष से कम के बच्चों की संख्या	___ ___ persons		
**7.** Single or joint household?<br>एकल या संयुक्त घर	Single ,dy ☐	Joint l;qä ☐	
**8.** Does anyone who lives in this household regularly smoke cigarettes, bidis, hukkahs or other tobacco products inside the home? क्या इस घर में रहने वाला कोई व्यक्ति सिगरेट, बीड़ी या हुक्का ?kj ds vUnj नियमित रूप से पीता है?	Yes gk ☐	No ugh ☐	Don't know/ Refused ekywe ugh@euk ☐
**9.** Is ANY of the following fuels used to cook food in hosuehold: coal/charcoal/dung cakes/wood / grass/straw / shrubs/ grass / agricultural crop waste?<br>क्या घर में खाना पकाने के लिये निम्नलिखित किसी इंधन का उपयोग किया जाता है: कोयला, झाड़ी घांस या भूसा, कृषीय फसल अपशिष्ट, गोबर के उपले, लकड़ी	Yes gk ☐	No ugh ☐	Don't know/ Refused ekywe ugh@euk ☐

IF RESPONSE TO QUESTION 9 IS "YES", COMPLETE QUESTION 10. ;fn Á'u 9 dk Tkokc ;g gS ßgkß Rk® Á'u 10 iNsA

IF RESPONSE TO QUESTION 9 IS "NO" OR "DON'T KNOW / REFUSED" , GO DIRECTLY TO QUESTION 14.
;fn Á'u 9 dk Tkokc ;g gS ßukß ;k Þekywe ughß Rk® lh/ks Á'u 14 iNsA

Influenza Disease Burden in India

**10.** What type of stove is usually used for cooking?(Probe for Type: Refer to photograph) घर में खाना पकाने के लिये किस प्रकार का स्टोव / चुल्हा प्रयोग किया जाता है ? (तस्वीर देखें)		
☐ Open fire	☐ Improved single-pot stove	☐ Griddle stove
☐ Surrounded fire	☐ Improved multi-pot stove	☐ Other (specify):

**11.** Is smoke removed by a chimney or a hood?<br>क्या धुंऐ को किसी चिमनी या हुड द्वारा हटाने का प्रबन्ध है ?	Chimney<br>चिमनी ☐	Hood<br>हुड ☐	Neither<br>कोई नहीं ☐

**12.** Is the cooking usually done in the indoor living space, in a separate kitchen/building, or outdoors?<br>अधिकतर खाना कहां बनता है?	☐ In a room used for living/sleeping.उस कमरे में जहां परिवार के सदस्य सोते भी हैं
	☐ In a separate room used as a kitchen. घर में एक अलग कमरा रसोई है
	☐ In a separate building used as a kitchen. अलग बिल्डिंग है जिसमें रसोई है
	☐ Outdoors खुले में
	☐ Other (specify): अन्य जगह पर (उल्लेख करें):

**13.** What type of ventilation is present where the stove is used?<br>जिस जगह स्टोव चुल्हा इस्तेमाल होता है वहां हवा के आर–पार जाने की क्या व्यवस्था है ?	☐ Closed room सभी खिड़की दरवाजे बंद रहते हैं
	☐ Room with eave spaces सभी खिड़की दरवाजे बंद रहते हैं लेकिन दीवार में हवा के लिये छेद हैं
	☐ Room with open windows/door कमरे में खिड़की या दरवाजे खुले रखे जाते हैं
	☐ Room with three or fewer walls कमरे में एक या अधिक दीवार नहीं है
	☐ Use the Stove Outdoors खुले में स्टोव चुल्हा इस्तेमाल होता है
	☐ Other (specify): अन्य (उल्लेख करें):

**14.** How is the cross-ventilation in the living rooms (i.e the rooms that are being used for daily activities and sleeping) **as reported** by the family members?<br>घर के जो कमरे काम करने या सोने के लिये इस्तेमाल होते है, उन कमरों में हवा के आर–पार जाने की क्या व्यवस्था है	☐ Adequate cross ventilation in all rooms<br>सभी कमरों में हवा के आर–पार जाने की पर्याप्त व्यवस्था है
	☐ Adequate crossventilation in some rooms<br>कुछ कमरों में हवा के आर–पार जाने की पर्याप्त व्यवस्था है
	☐ No room has adequate cross-ventilation<br>किसी भी कमरे में हवा के आर–पार जाने की पर्याप्त व्यवस्था नहीं है
	☐ No response जवाब नहीं

Adequacy means two or more door/windows opposite to each other.
dejk e gok d vkj&ikj tku dh Ik;kIr 0;oLFkk gku dk eryc g fd nk njokt ;k f[kMdh vkeu lkeu ¼dh nhokj ij½ g

**15.** What type of fuel does the household mainly use for heating?<br>घर को गर्म रखने के लिये किस इंधन का उपयोग किया जाता है ?	
☐ Kerosene मिट्टी का तेल	☐ Electricity बिजली
☐ Bio gas बायोगैस	☐ Other (specify):<br>अन्य (उल्लेख करें):
☐ LPG gas stove गैस का सिलेंडर	☐ None कुछ नहीं
☐ Dung cakes गोबर के उपले	

FAMILY: ___/____(New family number / Total number of new families) HOUSEHOLD HDSS ID: [1] PROJECT [ ] PHC No. [ ][ ] VILLAGE No. [ ][ ][ ][ ] House No.

Influenza Disease Burden in India

Proforma 2: Individual Data Sheet

Ask the number of members currently living at the household and the number of deaths in last 1 year. Then complete an individual data sheet for each household member, including those not enrolled in HDSS system and those who have died in the past year.

पता करें की घर में कितने सदस्य रहते हैं और पिछले 1 वर्ष में कितनी मृत्यु हुई हैं। फिर घर के सभी सदस्यों के लिये एक एक व्यक्तिगत शीट भरें। इसमें वे सदस्य भी शामलि हैं, जो HDSS प्रणाली में नामांकित नहीं हैं और जिनकी पिछले वर्ष मृत्यु हो गई है।

**Check if Key Respondent** ;fn e[; mRRkjnk;h g rk fu'kku yxk,	☐
1. HDSS ID Number:	X - X - X X - X X X X - ☐ ☐ - ☐ ☐
2. First Name नाम का पहला हिस्सा :	
3. Last Name नाम का अंतिम हिस्सा :	
4. Age उम्र:	Years ___ ___ ___ Months ___ ___ (1-12)

Pre-existing Health Conditions

For each problem that is indicated, complete question regarding treatment/medication. Indicate all that apply. बताई गई प्रत्येक बिमारी के संबंध में और उसके उपचार की स्थिति के बारे में पूछें। उन सभी बातों का उल्लेख करें, जो लागू हों।

Has this person ever been told by a healthcare provider that they have any of the following health conditions? क्या कभी किसी स्वास्थ्य कर्मी ने इन्हें निम्नलिखित में से कोई बीमारी बतायी है?			
HEALTH CONDITION बिमारी	Currently receiving treatment or taking medication for this condition? क्या अभी इस बिमारी का इलाज चल रहा है?		
☐ This individual has not been told they have any of the problems listed below. इस व्यक्ति को यह नहीं बताया कि सूची में दी गई बीमारियों में से उसे कोई बीमारी है।			
☐ Diabetes मधुमेह /शुगर	Yes हां ☐	No नहीं ☐	Don't know मालूम नहीं ☐
☐ Asthma दमा	Yes हां ☐	No नहीं ☐	Don't know मालूम नहीं ☐
☐ Chronic lung disease चिरकालिक फेफड़े का रोग	Yes हां ☐	No नहीं ☐	Don't know मालूम नहीं ☐
☐ Heart Condition हृदय रोग	Yes हां ☐	No नहीं ☐	Don't know मालूम नहीं ☐
☐ Stroke/CVA आघात/सीवीए	Yes हां ☐	No नहीं ☐	Don't know मालूम नहीं ☐
☐ Chronic liver disease चिरकालिक यकृत(लिवर) रोग	Yes हां ☐	No नहीं ☐	Don't know मालूम नहीं ☐
☐ HIV/AIDS एचआईवी/एड्स	Yes हां ☐	No नहीं ☐	Don't know मालूम नहीं ☐
☐ Chronic Kidney disease चिरकालिक गुर्दे का रोग	Yes हां ☐	No नहीं ☐	Don't know मालूम नहीं ☐
☐ Tuberculosis टी.बी.	Yes हां ☐	No नहीं ☐	Don't know मालूम नहीं ☐
☐ Neurological condition तंत्रिका संबंधी बीमारी	Yes हां ☐	No नहीं ☐	Don't know मालूम नहीं ☐
☐ High Blood Pressure उच्च रक्तचाप	Yes हां ☐	No नहीं ☐	Don't know मालूम नहीं ☐
☐ Other (specify below): अन्य (नीचे बताएं):	Yes हां ☐	No नहीं ☐	Don't know मालूम नहीं ☐

FAMILY: ___/____(New family number / Total number of new families) HOUSEHOLD HDSS ID: [1] PROJECT [ ] PHC No. [ ][ ] VILLAGE No. [ ][ ][ ][ ] House No.

Influenza Disease Burden in India

Check if Key Respondent ;fn e[; mRRkjnk;h g rk fu'kku yxk, ☐	
1. HDSS ID Number:	x - x - x x - x x x x - _ _ - _ _
2. First Name नाम का पहला हिस्सा :	
3. Last Name नाम का अंतिम हिस्सा :	
4. Age उम्र:	Years ___ ___ ___ Months ___ ___ (1-12)

Pre-existing Health Conditions

For each problem that is indicated, complete question regarding treatment/medication. Indicate all that apply. बताई गई प्रत्येक बिमारी के संबंध में और उसके उपचार की स्थिति के बारे में पूछें। उन सभी बातों का उल्लेख करें, जो लागू हों।

Has this person ever been told by a healthcare provider that they have any of the following health conditions? क्या कभी किसी स्वास्थ्य कर्मी ने इन्हें निम्नलिखित में से कोई बीमारी बतायी है?			
HEALTH CONDITION बिमारी	Currently receiving treatment or taking medication for this condition? क्या अभी इस बिमारी का इलाज चल रहा है?		
☐ This individual has not been told they have any of the problems listed below. इस व्यक्ति को यह नहीं बताया कि सूची में दी गई बीमारियों में से उसे कोई बीमारी है।			
☐ Diabetes मधुमेह /शुगर	Yes हां ☐	No नहीं ☐	Don't know मालूम नहीं ☐
☐ Asthma दमा	Yes हां ☐	No नहीं ☐	Don't know मालूम नहीं ☐
☐ Chronic lung disease चिरकालिक फेफड़े का रोग	Yes हां ☐	No नहीं ☐	Don't know मालूम नहीं ☐
☐ Heart Condition हृदय रोग	Yes हां ☐	No नहीं ☐	Don't know मालूम नहीं ☐
☐ Stroke/CVA आघात/सीवीए	Yes हां ☐	No नहीं ☐	Don't know मालूम नहीं ☐
☐ Chronic liver disease चिरकालिक यकृत(लिवर) रोग	Yes हां ☐	No नहीं ☐	Don't know मालूम नहीं ☐
☐ HIV/AIDS एचआईवी/एड्स	Yes हां ☐	No नहीं ☐	Don't know मालूम नहीं ☐
☐ Chronic Kidney disease चिरकालिक गुर्दे का रोग	Yes हां ☐	No नहीं ☐	Don't know मालूम नहीं ☐
☐ Tuberculosis टी.बी.	Yes हां ☐	No नहीं ☐	Don't know मालूम नहीं ☐
☐ Neurological condition तंत्रिका संबंधी बीमारी	Yes हां ☐	No नहीं ☐	Don't know मालूम नहीं ☐
☐ High Blood Pressure उच्च रक्तचाप	Yes हां ☐	No नहीं ☐	Don't know मालूम नहीं ☐
☐ Other (specify below): अन्य (नीचे बताएं):	Yes हां ☐	No नहीं ☐	Don't know मालूम नहीं ☐

Influenza Disease Burden in India

**Check if Key Respondent** ;fn e[; mRRkjnk;h g rk fu'kku yxk, ☐	
1. HDSS ID Number:	X - X - X X - X X X X - _ _ - _ _
2. First Name नाम का पहला हिस्सा :	
3. Last Name नाम का अंतिम हिस्सा :	
4. Age उम्र:	Years ___ ___ ___ Months ___ ___ (1-12)

Pre-existing Health Conditions

For each problem that is indicated, complete question regarding treatment/medication. Indicate all that apply. बताई गई प्रत्येक बिमारी के संबंध में और उसके उपचार की स्थिति के बारे में पूछें। उन सभी बातों का उल्लेख करें, जो लागू हों।

Has this person ever been told by a healthcare provider that they have any of the following health conditions? क्या कभी किसी स्वास्थ्य कर्मी ने इन्हें निम्नलिखित में से कोई बीमारी बतायी है?

HEALTH CONDITION बिमारी	Currently receiving treatment or taking medication for this condition? क्या अभी इस बिमारी का इलाज चल रहा है?		
☐ This individual has not been told they have any of the problems listed below. इस व्यक्ति को यह नहीं बताया कि सूची में दी गई बीमारियों में से उसे कोई बीमारी है।			
☐ Diabetes मधुमेह /शुगर	Yes हां ☐	No नहीं ☐	Don't know मालूम नहीं ☐
☐ Asthma दमा	Yes हां ☐	No नहीं ☐	Don't know मालूम नहीं ☐
☐ Chronic lung disease चिरकालिक फेफड़े का रोग	Yes हां ☐	No नहीं ☐	Don't know मालूम नहीं ☐
☐ Heart Condition हृदय रोग	Yes हां ☐	No नहीं ☐	Don't know मालूम नहीं ☐
☐ Stroke/CVA आघात/सीवीए	Yes हां ☐	No नहीं ☐	Don't know मालूम नहीं ☐
☐ Chronic liver disease चिरकालिक यकृत(लिवर) रोग	Yes हां ☐	No नहीं ☐	Don't know मालूम नहीं ☐
☐ HIV/AIDS एचआईवी/एड्स	Yes हां ☐	No नहीं ☐	Don't know मालूम नहीं ☐
☐ Chronic Kidney disease चिरकालिक गुर्दे का रोग	Yes हां ☐	No नहीं ☐	Don't know मालूम नहीं ☐
☐ Tuberculosis टी.बी.	Yes हां ☐	No नहीं ☐	Don't know मालूम नहीं ☐
☐ Neurological condition तंत्रिका संबंधी बीमारी	Yes हां ☐	No नहीं ☐	Don't know मालूम नहीं ☐
☐ High Blood Pressure उच्च रक्तचाप	Yes हां ☐	No नहीं ☐	Don't know मालूम नहीं ☐
☐ Other (specify below): अन्य (नीचे बताएं):	Yes हां ☐	No नहीं ☐	Don't know मालूम नहीं ☐

FAMILY: ___/____(New family number / Total number of new families) HOUSEHOLD HDSS ID: 1 PROJECT ☐ PHC No. ☐☐ VILLAGE No. ☐☐☐☐ House No.

Influenza Disease Burden in India

Check if Key Respondent ;fn e[; mRRkjnk;h g rk fu'kku yxk, ☐	
1. HDSS ID Number:	x - x - x x - x x x x - ☐☐ - ☐☐
2. First Name नाम का पहला हिस्सा :	
3. Last Name नाम का अंतिम हिस्सा :	
4. Age उम्र:	Years ___ ___ ___ Months ___ ___ (1-12)

Pre-existing Health Conditions

For each problem that is indicated, complete question regarding treatment/medication. Indicate all that apply. बताई गई प्रत्येक बिमारी के संबंध में और उसके उपचार की स्थिति के बारे में पूछें। उन सभी बातों का उल्लेख करें, जो लागू हों।

Has this person ever been told by a healthcare provider that they have any of the following health conditions? क्या कभी किसी स्वास्थ्य कर्मी ने इन्हें निम्नलिखित में से कोई बीमारी बतायी है?			
HEALTH CONDITION बिमारी	Currently receiving treatment or taking medication for this condition? क्या अभी इस बिमारी का इलाज चल रहा है?		
☐ This individual has not been told they have any of the problems listed below. इस व्यक्ति को यह नहीं बताया कि सूची में दी गई बीमारियों में से उसे कोई बीमारी है।			
☐ Diabetes मधुमेह /शुगर	Yes हां ☐	No नहीं ☐	Don't know मालूम नहीं ☐
☐ Asthma दमा	Yes हां ☐	No नहीं ☐	Don't know मालूम नहीं ☐
☐ Chronic lung disease चिरकालिक फेफड़े का रोग	Yes हां ☐	No नहीं ☐	Don't know मालूम नहीं ☐
☐ Heart Condition हृदय रोग	Yes हां ☐	No नहीं ☐	Don't know मालूम नहीं ☐
☐ Stroke/CVA आघात/सीवीए	Yes हां ☐	No नहीं ☐	Don't know मालूम नहीं ☐
☐ Chronic liver disease चिरकालिक यकृत(लिवर) रोग	Yes हां ☐	No नहीं ☐	Don't know मालूम नहीं ☐
☐ HIV/AIDS एचआईवी/एड्स	Yes हां ☐	No नहीं ☐	Don't know मालूम नहीं ☐
☐ Chronic Kidney disease चिरकालिक गुर्दे का रोग	Yes हां ☐	No नहीं ☐	Don't know मालूम नहीं ☐
☐ Tuberculosis टी.बी.	Yes हां ☐	No नहीं ☐	Don't know मालूम नहीं ☐
☐ Neurological condition तंत्रिका संबंधी बीमारी	Yes हां ☐	No नहीं ☐	Don't know मालूम नहीं ☐
☐ High Blood Pressure उच्च रक्तचाप	Yes हां ☐	No नहीं ☐	Don't know मालूम नहीं ☐
☐ Other (specify below): अन्य (नीचे बताएं):	Yes हां ☐	No नहीं ☐	Don't know मालूम नहीं ☐

Influenza Disease Burden in India

**Check if Key Respondent** ;fn e[; mRRkjnk;h g rk fu'kku yxk,a ☐	
1. HDSS ID Number:	x - x - x x - x x x x - ☐ ☐ - ☐ ☐
2. First Name नाम का पहला हिस्सा :	
3. Last Name नाम का अंतिम हिस्सा :	
4. Age उम्र:	Years ___ ___ ___ Months ___ ___ (1-12)

Pre-existing Health Conditions

For each problem that is indicated, complete question regarding treatment/medication. Indicate all that apply. बताई गई प्रत्येक बिमारी के संबंध में और उसके उपचार की स्थिति के बारे में पूछें। उन सभी बातों का उल्लेख करें, जो लागू हों।

Has this person ever been told by a healthcare provider that they have any of the following health conditions?
क्या कभी किसी स्वास्थ्य कर्मी ने इन्हें निम्नलिखित में से कोई बीमारी बतायी है?

HEALTH CONDITION बिमारी	Currently receiving treatment or taking medication for this condition? क्या अभी इस बिमारी का इलाज चल रहा है?		
☐ This individual has not been told they have any of the problems listed below. इस व्यक्ति को यह नहीं बताया कि सूची में दी गई बीमारियों में से उसे कोई बीमारी है।			
☐ Diabetes मधुमेह /शुगर	Yes हां ☐	No नहीं ☐	Don’t know मालूम नहीं ☐
☐ Asthma दमा	Yes हां ☐	No नहीं ☐	Don’t know मालूम नहीं ☐
☐ Chronic lung disease चिरकालिक फेफड़े का रोग	Yes हां ☐	No नहीं ☐	Don’t know मालूम नहीं ☐
☐ Heart Condition हृदय रोग	Yes हां ☐	No नहीं ☐	Don’t know मालूम नहीं ☐
☐ Stroke/CVA आघात/सीवीए	Yes हां ☐	No नहीं ☐	Don’t know मालूम नहीं ☐
☐ Chronic liver disease चिरकालिक यकृत(लिवर) रोग	Yes हां ☐	No नहीं ☐	Don’t know मालूम नहीं ☐
☐ HIV/AIDS एचआईवी/एड्स	Yes हां ☐	No नहीं ☐	Don’t know मालूम नहीं ☐
☐ Chronic Kidney disease चिरकालिक गुर्दे का रोग	Yes हां ☐	No नहीं ☐	Don’t know मालूम नहीं ☐
☐ Tuberculosis टी.बी.	Yes हां ☐	No नहीं ☐	Don’t know मालूम नहीं ☐
☐ Neurological condition तंत्रिका संबंधी बीमारी	Yes हां ☐	No नहीं ☐	Don’t know मालूम नहीं ☐
☐ High Blood Pressure उच्च रक्तचाप	Yes हां ☐	No नहीं ☐	Don’t know मालूम नहीं ☐
☐ Other (specify below): अन्य (नीचे बताएं):	Yes हां ☐	No नहीं ☐	Don’t know मालूम नहीं ☐

FAMILY: ___/____(New family number / Total number of new families) HOUSEHOLD HDSS ID: [1] PROJECT [ ] PHC No. [ ][ ] VILLAGE No. [ ][ ][ ][ ] House No.

Influenza Disease Burden in India

**Check if Key Respondent** ;fn e[; mRRkjnk;h g rk fu'kku yxk,	☐
1. HDSS ID Number:	x - x - x x - x x x x - _ _ - _ _
2. First Name नाम का पहला हिस्सा :	
3. Last Name नाम का अंतिम हिस्सा :	
4. Age उम्र:	Years ___ ___ ___ Months ___ ___ (1-12)

Pre-existing Health Conditions

For each problem that is indicated, complete question regarding treatment/medication. Indicate all that apply. बताई गई प्रत्येक बिमारी के संबंध में और उसके उपचार की स्थिति के बारे में पूछें। उन सभी बातों का उल्लेख करें, जो लागू हों।

Has this person ever been told by a healthcare provider that they have any of the following health conditions? क्या कभी किसी स्वास्थ्य कर्मी ने इन्हें निम्नलिखित में से कोई बीमारी बतायी है?

HEALTH CONDITION बिमारी	Currently receiving treatment or taking medication for this condition? क्या अभी इस बिमारी का इलाज चल रहा है?		
☐ This individual has not been told they have any of the problems listed below. इस व्यक्ति को यह नहीं बताया कि सूची में दी गई बीमारियों में से उसे कोई बीमारी है।			
☐ Diabetes मधुमेह / शुगर	Yes हां ☐	No नहीं ☐	Don't know मालूम नहीं ☐
☐ Asthma दमा	Yes हां ☐	No नहीं ☐	Don't know मालूम नहीं ☐
☐ Chronic lung disease चिरकालिक फेफड़े का रोग	Yes हां ☐	No नहीं ☐	Don't know मालूम नहीं ☐
☐ Heart Condition हृदय रोग	Yes हां ☐	No नहीं ☐	Don't know मालूम नहीं ☐
☐ Stroke/CVA आघात / सीवीए	Yes हां ☐	No नहीं ☐	Don't know मालूम नहीं ☐
☐ Chronic liver disease चिरकालिक यकृत(लिवर) रोग	Yes हां ☐	No नहीं ☐	Don't know मालूम नहीं ☐
☐ HIV/AIDS एचआईवी / एड्स	Yes हां ☐	No नहीं ☐	Don't know मालूम नहीं ☐
☐ Chronic Kidney disease चिरकालिक गुर्दे का रोग	Yes हां ☐	No नहीं ☐	Don't know मालूम नहीं ☐
☐ Tuberculosis टी.बी.	Yes हां ☐	No नहीं ☐	Don't know मालूम नहीं ☐
☐ Neurological condition तंत्रिका संबंधी बीमारी	Yes हां ☐	No नहीं ☐	Don't know मालूम नहीं ☐
☐ High Blood Pressure उच्च रक्तचाप	Yes हां ☐	No नहीं ☐	Don't know मालूम नहीं ☐
☐ Other (specify below): अन्य (नीचे बताएं):	Yes हां ☐	No नहीं ☐	Don't know मालूम नहीं ☐

Influenza Disease Burden in India

**Check if Key Respondent** ;fn e[; mRRkjnk;h gS rks fu'kku yxk,¡	☐
1. HDSS ID Number:	X - X - X X - X X X X - ☐ ☐ - ☐ ☐
2. First Name नाम का पहला हिस्सा :	
3. Last Name नाम का अंतिम हिस्सा :	
4. Age उम्र:	Years ___ ___ ___ Months ___ ___ (1-12)

Pre-existing Health Conditions

For each problem that is indicated, complete question regarding treatment/medication. Indicate all that apply. बताई गई प्रत्येक बिमारी के संबंध में और उसके उपचार की स्थिति के बारे में पूछें। उन सभी बातों का उल्लेख करें, जो लागू हों।

Has this person ever been told by a healthcare provider that they have any of the following health conditions?
क्या कभी किसी स्वास्थ्य कर्मी ने इन्हें निम्नलिखित में से कोई बीमारी बतायी है?

HEALTH CONDITION बिमारी	Currently receiving treatment or taking medication for this condition? क्या अभी इस बिमारी का इलाज चल रहा है?		
☐ This individual has not been told they have any of the problems listed below. इस व्यक्ति को यह नहीं बताया कि सूची में दी गई बीमारियों में से उसे कोई बीमारी है।			
☐ Diabetes मधुमेह /शुगर	Yes हां ☐	No नहीं ☐	Don't know मालूम नहीं ☐
☐ Asthma दमा	Yes हां ☐	No नहीं ☐	Don't know मालूम नहीं ☐
☐ Chronic lung disease चिरकालिक फेफड़े का रोग	Yes हां ☐	No नहीं ☐	Don't know मालूम नहीं ☐
☐ Heart Condition हृदय रोग	Yes हां ☐	No नहीं ☐	Don't know मालूम नहीं ☐
☐ Stroke/CVA आघात/सीवीए	Yes हां ☐	No नहीं ☐	Don't know मालूम नहीं ☐
☐ Chronic liver disease चिरकालिक यकृत(लिवर) रोग	Yes हां ☐	No नहीं ☐	Don't know मालूम नहीं ☐
☐ HIV/AIDS एचआईवी/एड्स	Yes हां ☐	No नहीं ☐	Don't know मालूम नहीं ☐
☐ Chronic Kidney disease चिरकालिक गुर्दे का रोग	Yes हां ☐	No नहीं ☐	Don't know मालूम नहीं ☐
☐ Tuberculosis टी.बी.	Yes हां ☐	No नहीं ☐	Don't know मालूम नहीं ☐
☐ Neurological condition तंत्रिका संबंधी बीमारी	Yes हां ☐	No नहीं ☐	Don't know मालूम नहीं ☐
☐ High Blood Pressure उच्च रक्तचाप	Yes हां ☐	No नहीं ☐	Don't know मालूम नहीं ☐
☐ Other (specify below): अन्य (नीचे बताएं):	Yes हां ☐	No नहीं ☐	Don't know मालूम नहीं ☐

FAMILY: ___/____(New family number / Total number of new families) HOUSEHOLD HDSS ID: [1] PROJECT [ ] PHC No. [ ][ ] VILLAGE No. [ ][ ][ ][ ] House No.

Influenza Disease Burden in India

Check if Key Respondent ;fn e[; mRRkjnk;h g rk fu'kku yxk, ☐	
1. HDSS ID Number:	x - x - x x - x x x x - ☐☐ - ☐☐
2. First Name नाम का पहला हिस्सा :	
3. Last Name नाम का अंतिम हिस्सा :	
4. Age उम्र:	Years ___ ___ ___ Months ___ ___ (1-12)

Pre-existing Health Conditions

For each problem that is indicated, complete question regarding treatment/medication. Indicate all that apply. बताई गई प्रत्येक बिमारी के संबंध में और उसके उपचार की स्थिति के बारे में पूछें। उन सभी बातों का उल्लेख करें, जो लागू हों।

Has this person ever been told by a healthcare provider that they have any of the following health conditions?
क्या कभी किसी स्वास्थ्य कर्मी ने इन्हें निम्नलिखित में से कोई बीमारी बतायी है?

HEALTH CONDITION बिमारी	Currently receiving treatment or taking medication for this condition? क्या अभी इस बिमारी का इलाज चल रहा है?		
☐ This individual has not been told they have any of the problems listed below. इस व्यक्ति को यह नहीं बताया कि सूची में दी गई बीमारियों में से उसे कोई बीमारी है।			
☐ Diabetes मधुमेह / शुगर	Yes हां ☐	No नहीं ☐	Don't know मालूम नहीं ☐
☐ Asthma दमा	Yes हां ☐	No नहीं ☐	Don't know मालूम नहीं ☐
☐ Chronic lung disease चिरकालिक फेफड़े का रोग	Yes हां ☐	No नहीं ☐	Don't know मालूम नहीं ☐
☐ Heart Condition हृदय रोग	Yes हां ☐	No नहीं ☐	Don't know मालूम नहीं ☐
☐ Stroke/CVA आघात / सीवीए	Yes हां ☐	No नहीं ☐	Don't know मालूम नहीं ☐
☐ Chronic liver disease चिरकालिक यकृत(लिवर) रोग	Yes हां ☐	No नहीं ☐	Don't know मालूम नहीं ☐
☐ HIV/AIDS एचआईवी / एड्स	Yes हां ☐	No नहीं ☐	Don't know मालूम नहीं ☐
☐ Chronic Kidney disease चिरकालिक गुर्दे का रोग	Yes हां ☐	No नहीं ☐	Don't know मालूम नहीं ☐
☐ Tuberculosis टी.बी.	Yes हां ☐	No नहीं ☐	Don't know मालूम नहीं ☐
☐ Neurological condition तंत्रिका संबंधी बीमारी	Yes हां ☐	No नहीं ☐	Don't know मालूम नहीं ☐
☐ High Blood Pressure उच्च रक्तचाप	Yes हां ☐	No नहीं ☐	Don't know मालूम नहीं ☐
☐ Other (specify below): अन्य (नीचे बताएं):	Yes हां ☐	No नहीं ☐	Don't know मालूम नहीं ☐

Influenza Disease Burden in India

**Check if Key Respondent** ;fn e[; mRRkjnk;h g rk fu'kku yxk, ☐	
1. HDSS ID Number:	x - x - x x - x x x x - _ _ - _ _
2. First Name नाम का पहला हिस्सा :	
3. Last Name नाम का अंतिम हिस्सा :	
4. Age उम्र:	Years ___ ___ ___ Months ___ ___ (1-12)

Pre-existing Health Conditions

For each problem that is indicated, complete question regarding treatment/medication. Indicate all that apply. बताई गई प्रत्येक बिमारी के संबंध में और उसके उपचार की स्थिति के बारे में पूछें। उन सभी बातों का उल्लेख करें, जो लागू हों।

Has this person ever been told by a healthcare provider that they have any of the following health conditions? क्या कभी किसी स्वास्थ्य कर्मी ने इन्हें निम्नलिखित में से कोई बीमारी बतायी है?			
HEALTH CONDITION बिमारी	Currently receiving treatment or taking medication for this condition? क्या अभी इस बिमारी का इलाज चल रहा है?		
☐ This individual has not been told they have any of the problems listed below. इस व्यक्ति को यह नहीं बताया कि सूची में दी गई बीमारियों में से उसे कोई बीमारी है।			
☐ Diabetes मधुमेह /शुगर	Yes हां ☐	No नहीं ☐	Don't know मालूम नहीं ☐
☐ Asthma दमा	Yes हां ☐	No नहीं ☐	Don't know मालूम नहीं ☐
☐ Chronic lung disease चिरकालिक फेफड़े का रोग	Yes हां ☐	No नहीं ☐	Don't know मालूम नहीं ☐
☐ Heart Condition हृदय रोग	Yes हां ☐	No नहीं ☐	Don't know मालूम नहीं ☐
☐ Stroke/CVA आघात/सीवीए	Yes हां ☐	No नहीं ☐	Don't know मालूम नहीं ☐
☐ Chronic liver disease चिरकालिक यकृत(लिवर) रोग	Yes हां ☐	No नहीं ☐	Don't know मालूम नहीं ☐
☐ HIV/AIDS एचआईवी/एड्स	Yes हां ☐	No नहीं ☐	Don't know मालूम नहीं ☐
☐ Chronic Kidney disease चिरकालिक गुर्दे का रोग	Yes हां ☐	No नहीं ☐	Don't know मालूम नहीं ☐
☐ Tuberculosis टी.बी.	Yes हां ☐	No नहीं ☐	Don't know मालूम नहीं ☐
☐ Neurological condition तंत्रिका संबंधी बीमारी	Yes हां ☐	No नहीं ☐	Don't know मालूम नहीं ☐
☐ High Blood Pressure उच्च रक्तचाप	Yes हां ☐	No नहीं ☐	Don't know मालूम नहीं ☐
☐ Other (specify below): अन्य (नीचे बताएं):	Yes हां ☐	No नहीं ☐	Don't know मालूम नहीं ☐

Influenza Disease Burden in India

Check if Key Respondent ;fn e[; mRRkjnk;h gS rks fu'kku yxk,a ☐	
1. HDSS ID Number:	X - X - X X - X X X X - __ __ - __ __
2. First Name नाम का पहला हिस्सा :	
3. Last Name नाम का अंतिम हिस्सा :	
4. Age उम्र:	Years ___ ___ ___ Months ___ ___ (1-12)

Pre-existing Health Conditions

For each problem that is indicated, complete question regarding treatment/medication. Indicate all that apply. बताई गई प्रत्येक बिमारी के संबंध में और उसके उपचार की स्थिति के बारे में पूछें। उन सभी बातों का उल्लेख करें, जो लागू हों।

Has this person ever been told by a healthcare provider that they have any of the following health conditions? क्या कभी किसी स्वास्थ्य कर्मी ने इन्हें निम्नलिखित में से कोई बीमारी बतायी है?

HEALTH CONDITION बिमारी	Currently receiving treatment or taking medication for this condition? क्या अभी इस बिमारी का इलाज चल रहा है?		
☐ This individual has not been told they have any of the problems listed below. इस व्यक्ति को यह नहीं बताया कि सूची में दी गई बीमारियों में से उसे कोई बीमारी है।			
☐ Diabetes मधुमेह /शुगर	Yes हां ☐	No नहीं ☐	Don't know मालूम नहीं ☐
☐ Asthma दमा	Yes हां ☐	No नहीं ☐	Don't know मालूम नहीं ☐
☐ Chronic lung disease चिरकालिक फेफड़े का रोग	Yes हां ☐	No नहीं ☐	Don't know मालूम नहीं ☐
☐ Heart Condition हृदय रोग	Yes हां ☐	No नहीं ☐	Don't know मालूम नहीं ☐
☐ Stroke/CVA आघात/सीवीए	Yes हां ☐	No नहीं ☐	Don't know मालूम नहीं ☐
☐ Chronic liver disease चिरकालिक यकृत(लिवर) रोग	Yes हां ☐	No नहीं ☐	Don't know मालूम नहीं ☐
☐ HIV/AIDS एचआईवी/एड्स	Yes हां ☐	No नहीं ☐	Don't know मालूम नहीं ☐
☐ Chronic Kidney disease चिरकालिक गुर्दे का रोग	Yes हां ☐	No नहीं ☐	Don't know मालूम नहीं ☐
☐ Tuberculosis टी.बी.	Yes हां ☐	No नहीं ☐	Don't know मालूम नहीं ☐
☐ Neurological condition तंत्रिका संबंधी बीमारी	Yes हां ☐	No नहीं ☐	Don't know मालूम नहीं ☐
☐ High Blood Pressure उच्च रक्तचाप	Yes हां ☐	No नहीं ☐	Don't know मालूम नहीं ☐
☐ Other (specify below): अन्य (नीचे बताएं):	Yes हां ☐	No नहीं ☐	Don't know मालूम नहीं ☐

Influenza Disease Burden in India

**Check if Key Respondent** ;fn e[; mRRkjnk;h g rk fu'kku yxk,¡ ☐	
1. HDSS ID Number:	x - x - x x - x x x x - ☐☐ - ☐☐
2. First Name नाम का पहला हिस्सा :	
3. Last Name नाम का अंतिम हिस्सा :	
4. Age उम्र:	Years ___ ___ ___ Months ___ ___ (1-12)

Pre-existing Health Conditions

For each problem that is indicated, complete question regarding treatment/medication. Indicate all that apply. बताई गई प्रत्येक बिमारी के संबंध में और उसके उपचार की स्थिति के बारे में पूछें। उन सभी बातों का उल्लेख करें, जो लागू हों।

Has this person ever been told by a healthcare provider that they have any of the following health conditions? क्या कभी किसी स्वास्थ्य कर्मी ने इन्हें निम्नलिखित में से कोई बीमारी बतायी है?

HEALTH CONDITION बिमारी	Currently receiving treatment or taking medication for this condition? क्या अभी इस बिमारी का इलाज चल रहा है?		
☐ This individual has not been told they have any of the problems listed below. इस व्यक्ति को यह नहीं बताया कि सूची में दी गई बीमारियों में से उसे कोई बीमारी है।			
☐ Diabetes मधुमेह / शुगर	Yes हां ☐	No नहीं ☐	Don't know मालूम नहीं ☐
☐ Asthma दमा	Yes हां ☐	No नहीं ☐	Don't know मालूम नहीं ☐
☐ Chronic lung disease चिरकालिक फेफड़े का रोग	Yes हां ☐	No नहीं ☐	Don't know मालूम नहीं ☐
☐ Heart Condition हृदय रोग	Yes हां ☐	No नहीं ☐	Don't know मालूम नहीं ☐
☐ Stroke/CVA आघात / सीवीए	Yes हां ☐	No नहीं ☐	Don't know मालूम नहीं ☐
☐ Chronic liver disease चिरकालिक यकृत(लिवर) रोग	Yes हां ☐	No नहीं ☐	Don't know मालूम नहीं ☐
☐ HIV/AIDS एचआईवी / एड्स	Yes हां ☐	No नहीं ☐	Don't know मालूम नहीं ☐
☐ Chronic Kidney disease चिरकालिक गुर्दे का रोग	Yes हां ☐	No नहीं ☐	Don't know मालूम नहीं ☐
☐ Tuberculosis टी.बी.	Yes हां ☐	No नहीं ☐	Don't know मालूम नहीं ☐
☐ Neurological condition तंत्रिका संबंधी बीमारी	Yes हां ☐	No नहीं ☐	Don't know मालूम नहीं ☐
☐ High Blood Pressure उच्च रक्तचाप	Yes हां ☐	No नहीं ☐	Don't know मालूम नहीं ☐
☐ Other (specify below): अन्य (नीचे बताएं):	Yes हां ☐	No नहीं ☐	Don't know मालूम नहीं ☐

Influenza Disease Burden in India

**Check if Key Respondent** ;fn e[; mRRkjnk;h g rk fu'kku yxk, ☐	
1. HDSS ID Number:	X - X - X X - X X X X - ☐☐ - ☐☐
2. First Name नाम का पहला हिस्सा :	
3. Last Name नाम का अंतिम हिस्सा :	
4. Age उम्र:	Years ___ ___ ___ Months ___ ___ (1-12)

Pre-existing Health Conditions

For each problem that is indicated, complete question regarding treatment/medication. Indicate all that apply. बताई गई प्रत्येक बिमारी के संबंध में और उसके उपचार की स्थिति के बारे में पूछें। उन सभी बातों का उल्लेख करें, जो लागू हों।

Has this person ever been told by a healthcare provider that they have any of the following health conditions? क्या कभी किसी स्वास्थ्य कर्मी ने इन्हें निम्नलिखित में से कोई बीमारी बतायी है?			
HEALTH CONDITION बिमारी	Currently receiving treatment or taking medication for this condition? क्या अभी इस बिमारी का इलाज चल रहा है?		
☐ This individual has not been told they have any of the problems listed below. इस व्यक्ति को यह नहीं बताया कि सूची में दी गई बीमारियों में से उसे कोई बीमारी है।			
☐ Diabetes मधुमेह /शुगर	Yes हां ☐	No नहीं ☐	Don't know मालूम नहीं ☐
☐ Asthma दमा	Yes हां ☐	No नहीं ☐	Don't know मालूम नहीं ☐
☐ Chronic lung disease चिरकालिक फेफड़े का रोग	Yes हां ☐	No नहीं ☐	Don't know मालूम नहीं ☐
☐ Heart Condition हृदय रोग	Yes हां ☐	No नहीं ☐	Don't know मालूम नहीं ☐
☐ Stroke/CVA आघात/सीवीए	Yes हां ☐	No नहीं ☐	Don't know मालूम नहीं ☐
☐ Chronic liver disease चिरकालिक यकृत(लिवर) रोग	Yes हां ☐	No नहीं ☐	Don't know मालूम नहीं ☐
☐ HIV/AIDS एचआईवी/एड्स	Yes हां ☐	No नहीं ☐	Don't know मालूम नहीं ☐
☐ Chronic Kidney disease चिरकालिक गुर्दे का रोग	Yes हां ☐	No नहीं ☐	Don't know मालूम नहीं ☐
☐ Tuberculosis टी.बी.	Yes हां ☐	No नहीं ☐	Don't know मालूम नहीं ☐
☐ Neurological condition तंत्रिका संबंधी बीमारी	Yes हां ☐	No नहीं ☐	Don't know मालूम नहीं ☐
☐ High Blood Pressure उच्च रक्तचाप	Yes हां ☐	No नहीं ☐	Don't know मालूम नहीं ☐
☐ Other (specify below): अन्य (नीचे बताएं):	Yes हां ☐	No नहीं ☐	Don't know मालूम नहीं ☐

If there are more than 12 house members, then attach extra sheets to this proforma and mention here the number of extra sheets attached________________________

Comments related to location of house / family

Burden of Severe Influenza in Rural India

Proforma 3: Health Utilization Survey

Date Form Completed: ____ ____ / ____ ____ / ____ ____ ____ ____ (dd/mm/yyyy)

**Time of starting interview**		**Time of Finishing interview**	
**Data Collected By:**			

Part 1-A: Recent Hospitalizations

**1.** During the last 6 months has anyone in this household been admitted to a hospital for an overnight stay?<br>क्या इस घर में से कोई व्यक्ति पिछले 6 मास के दौरान कम से कम एक रात के लिए अस्पताल में भर्ती हुआ है?	Yes ☐	No ☐	Don't know ☐

NOTE TO INTERVIEWER: This may include any hospitalizations that required an overnight stay for any reason. This does not include visits to a hospital that did not result in an overnight stay (such as for outpatient services at a hospital facility).

साक्षात्कर्ता के लिए टिप्पणी: इसमें अस्पताल में भर्ती होने की ऐसी कोई घटना शामिल की जा सकती है, जिसमें किसी कारण से कम से कम एक रात अस्पताल में रहना पड़ा हो। इसमें वह स्थिति शामिल नहीं है, जिसमें अस्पताल में जाना होता है और उसके कारण रात में नहीं रुकना पड़ता है (जैसे अस्पताल में कोई बाहिय रोगी सेवाओं का लाभ उठाने जाता है)।

IF YES, COMPLETE QUESTION 2 (TABLE ON FOLLOWING PAGE)
;fn gk] rks iz'u 2¼vxys i"B dh rkfydk½ HkjsA

IF NO, SKIP TO <u>PART 2-A</u>
;fn ugha] rks <u>Hkkx 2&d</u> Hkjs

IF DON'T KNOW, MAKE ARRANGEMENTS TO RETURN WHEN APPROPRIATE INFORMANT IS AVAILABLE.
;fn ekywe ugha] rks lwfpr lwpuk miyC/k gksus ij nqckjk vkus dh 0;oLFkk djsA

NOTE TO INTERVIEWER: In the following table, record all hospitalizations that occurred during the last 6 months among members of this household. This may include multiple events among single household members. This should include any hospitalizations within the past six months among members of the household who have recently died.

साक्षात्कर्ता के लिए टिप्पणी: निम्नलिखित तालिका में इस घर के सदस्यों में से पिछले छह माह के दौरान अस्पताल में भर्ती होने की सभी घटनाओं को रिकार्ड करें। इसमें किसी एक सदस्य के बार–बार अस्पताल में भर्ती में होने की घटना भी शामिल है। इसमें इस घर के सदस्यों में से पिछले छह माह के दौरान किसी के अस्पताल में भर्ती होने की ऐसी घटना को भी शामिल करें, जिसकी हाल ही में मृत्यु हो गई हो।

FOR EACH HOSPITALIZATION DUE TO "ACUTE MEDICAL ILLNESS" OR "OTHER" IN QUESTION 3 , COMPLETE A SEPARATE <u>PART 1-B</u>. THIS SHOULD BEGIN WITH THE MOST RECENT HOSPITALIZATION AND THEN SEQUENTIALLY PROGRESS TO EARLIER HOSPITALIZATIONS.
iz'u 3 ÞvkdfLed fpfdRlh; chekjhß ;k ÞvU;ß ds dkj.k izR;sd ckj vLirky esa HkrhZ gksus ds lc/k esa ,d vyx <u>Hkkx 1&[k</u> HkjsA vLirky esa Hkrhz gksus dh gky dh ?kVuk ls 'kq: djds fiNys fnuks vLirky esa Hkrhz gksus dh ?kVukvksa dks HkjsA

ONCE <u>PART 1-B</u> IS COMPLETED FOR ALL RELEVANT HOSPITALIZATIONS, CONTINUE TO <u>PART 2-A.</u>
vLirky esa Hkrhz gksus dh lHkh lxr ?kVukvksa dks ,d ckj <u>Hkkx 1&[k</u> esa Hkj nsus ds i'pkr <u>Hkkx 2 &d</u> tkjh j[ksaA

FAMILY: ___/____(New family number / Total number of new families) HOUSEHOLD HDSS ID: [1] PROJECT [ ] PHC No. [ ][ ] VILLAGE No. [ ][ ][ ][ ] HOUSE No.

Burden of Severe Influenza in Rural India

2. Please provide the following details for each hospital stay, **beginning with most recent**:
कृपया अस्पताल में भर्ती होने की gky dh ?kVuk l 'k: djd प्रत्येक घटना के संबंध में निम्नलिखित विवरण दें।

Event	Person's Name & HDSS ID No vLirky e Hkrh 0;fä dk uke@,pMh,l,l l-	Age (Yr & mth) me ¼o"k vkj ekl½	Cause of Hospital Stay vLirky e Hkrh gku dk dkj.k	Hospital Code vLirky dk dkM	Name of Hospital (if code 21) & Type vLirky dk uke & idkj ¼;fn dkM 21 g½	Date of Admission (dd/mm/yyyy) Hkrh gku dh rkjh[k
1.	X - X - XX - XXXX – [ ][ ] - [ ][ ]	___ ___yrs<br>___ ___mos	☐ Accident or Injury दुर्घटना या चोट<br>☐ Obstetrical (Delivery, Problems with Childbirth) प्रसूति संबंधी (प्रसव, बच्चे के जन्म से संबंधित समस्या)<br>☐ Surgery or Scheduled Procedure शल्य चिकित्सा या अनुसूचित प्रक्रिया<br>☐ Acute Medical Illness गंभीर चिकित्सीय बीमारी<br>☐ Other **(specify):** अन्य ¼mYys[k djs½	___ ___	**Name:**<br>☐ Public सरकारी<br>☐ Private प्राइवेट<br>☐ NGO गैर सरकारी संस्थान<br>☐ Don't Know मालूम नहीं	__ __ / __ __ / __ __ __ __
2.	X - X - XX - XXXX – [ ][ ] - [ ][ ]	___ ___yrs<br>___ ___mos	☐ Accident or Injury दुर्घटना या चोट<br>☐ Obstetrical (Delivery, Problems with Childbirth) प्रसूति संबंधी (प्रसव, बच्चे के जन्म से संबंधित समस्या)<br>☐ Surgery or Scheduled Procedure शल्य चिकित्सा या अनुसूचित प्रक्रिया<br>☐ Acute Medical Illness गंभीर चिकित्सीय बीमारी<br>☐ Other **(specify):** अन्य ¼mYys[k djs½	___ ___	**Name:**<br>☐ Public सरकारी<br>☐ Private प्राइवेट<br>☐ NGO गैर सरकारी संस्थान<br>☐ Don't Know मालूम नहीं	__ __ / __ __ / __ __ __ __
3.	X - X - XX - XXXX – [ ][ ] - [ ][ ]	___ ___yrs<br>___ ___mos	☐ Accident or Injury दुर्घटना या चोट<br>☐ Obstetrical (Delivery, Problems with Childbirth) प्रसूति संबंधी (प्रसव, बच्चे के जन्म से संबंधित समस्या)<br>☐ Surgery or Scheduled Procedure शल्य चिकित्सा या अनुसूचित प्रक्रिया<br>☐ Acute Medical Illness गंभीर चिकित्सीय बीमारी<br>☐ Other **(specify):** अन्य ¼mYys[k djs½	___ ___	**Name:**<br>☐ Public सरकारी<br>☐ Private प्राइवेट<br>☐ NGO गैर सरकारी संस्थान<br>☐ Don't Know मालूम नहीं	__ __ / __ __ / __ __ __ __

CODES TO COMPLETE COLUMN "HOSPITAL CODE"

**01**	Ramesh Nursing Home	**05**	Bhatia Nursing Home	**09**	Jindal Nursing Home	**13**	Manju Shree Nursing Home	**17**	BK Hospital
**02**	Manish Hospital	**06**	Ballabgarh Nursing Home	**10**	Mittal Hospital	**14**	Sirohi Medical Center	**18**	Sun Flag Hospital
**03**	Arya Nursing Home	**07**	Kesar Nursing Home	**11**	Prakash Hospital	**15**	Akash Hospital	**19**	Escort Hospital
**04**	Dr. Sudarshan Gupta Hospital	**08**	Gupta Hospital	**12**	Keshav Nursing Home	**16**	Civil Hospital, Ballabgarh	**20**	AIIMS – Delhi **21** **Other (specify in table)**

FAMILY: ___/____(New family number / Total number of new families) HOUSEHOLD HDSS ID: [1] PROJECT [ ] PHC No. [ ][ ] VILLAGE No. [ ][ ][ ][ ] HOUSE No.

Burden of Severe Influenza in Rural India

4.	X - X - X X - X X X X –<br>[ ][ ] - [ ][ ]	____ ____yrs<br>____ ____mos	☐ Accident or Injury दुर्घटना या चोट<br>☐ Obstetrical (Delivery, Problems with Childbirth) प्रसूति संबंधी (प्रसव, बच्चे के जन्म से संबंधित समस्या)<br>☐ Surgery or Scheduled Procedure शल्य चिकित्सा या अनुसूचित प्रक्रिया<br>☐ Acute Medical Illness गंभीर चिकित्सीय बीमारी<br>☐ Other **(specify):** अन्य ¼mYy[k dj½	___ ___	**Name:**<br>☐ Public सरकारी<br>☐ Private प्राइवेट<br>☐ NGO गैर सरकारी संस्थान<br>☐ Don't Know मालूम नहीं	__ __ / __ __ /<br>__ __ __ __
5.	X - X - X X - X X X X –<br>[ ][ ] - [ ][ ]	____ ____yrs<br>____ ____mos	☐ Accident or Injury दुर्घटना या चोट<br>☐ Obstetrical (Delivery, Problems with Childbirth) प्रसूति संबंधी (प्रसव, बच्चे के जन्म से संबंधित समस्या)<br>☐ Surgery or Scheduled Procedure शल्य चिकित्सा या अनुसूचित प्रक्रिया<br>☐ Acute Medical Illness गंभीर चिकित्सीय बीमारी<br>☐ Other **(specify):** अन्य ¼mYy[k dj½	___ ___	**Name:**<br>☐ Public सरकारी<br>☐ Private प्राइवेट<br>☐ NGO गैर सरकारी संस्थान<br>☐ Don't Know मालूम नहीं	__ __ / __ __ /<br>__ __ __ __
6.	X - X - X X - X X X X –<br>[ ][ ] - [ ][ ]	____ ____yrs<br>____ ____mos	☐ Accident or Injury दुर्घटना या चोट<br>☐ Obstetrical (Delivery, Problems with Childbirth) प्रसूति संबंधी (प्रसव, बच्चे के जन्म से संबंधित समस्या)<br>☐ Surgery or Scheduled Procedure शल्य चिकित्सा या अनुसूचित प्रक्रिया<br>☐ Acute Medical Illness गंभीर चिकित्सीय बीमारी<br>☐ Other **(specify):** अन्य ¼mYy[k dj½	___ ___	**Name:**<br>☐ Public सरकारी<br>☐ Private प्राइवेट<br>☐ NGO गैर सरकारी संस्थान<br>☐ Don't Know मालूम नहीं	__ __ / __ __ /<br>__ __ __ __
7.	X - X - X X - X X X X –<br>[ ][ ] - [ ][ ]	____ ____yrs<br>____ ____mos	☐ Accident or Injury दुर्घटना या चोट<br>☐ Obstetrical (Delivery, Problems with Childbirth) प्रसूति संबंधी (प्रसव, बच्चे के जन्म से संबंधित समस्या)<br>☐ Surgery or Scheduled Procedure शल्य चिकित्सा या अनुसूचित प्रक्रिया<br>☐ Acute Medical Illness गंभीर चिकित्सीय बीमारी<br>☐ Other **(specify):** अन्य ¼mYy[k dj½	___ ___	**Name:**<br>☐ Public सरकारी<br>☐ Private प्राइवेट<br>☐ NGO गैर सरकारी संस्थान<br>☐ Don't Know मालूम नहीं	__ __ / __ __ /<br>__ __ __ __

FAMILY: ___/____(New family number / Total number of new families) HOUSEHOLD HDSS ID: [1] [ ] [ ][ ] [ ][ ][ ][ ]
PROJECT PHC No. VILLAGE No. HOUSE No.

Burden of Severe Influenza in Rural India

Proforma 3: Health Utilization Survey

Part 1-B: Hospitalization Proforma Hkkx 1&[k ½ vLirky izkQkekZ

COMPLETE THIS PROFORMA FOR EACH HOSPITALIZATION DUE TO "ACUTE MEDICAL ILLNESS" OR "OTHER" FROM HOSPITALIZATION TABLE IN PART 1-A

ÞxHkhj fpfdRlh; chekjhß ;k ÞvU;ß dh dkj.k iR;sd ckj vLirky esa Hkrhz gksus dh lwph/k esa bl izkQkekZ dks Hkkx 1&d esa nh xbZ vLirky esa Hkrhz gksus dh rkfydk ls HkjsA

1. Name of hospital where admitted: vLirky dk uke

☐ Ramesh Nursing Home	☐ Gupta Hospital	☐ Akash Hospital
☐ Manish Hospital	☐ Jindal Nursing Home	☐ Civil Hospital, Ballabgarh
☐ Arya Nursing Home	☐ Mittal Hospital	☐ BK Hospital
☐ Dr. Sudarshan Gupta Hospital	☐ Prakash Hospital	☐ Sun Flag Hospital
☐ Bhatia Nursing Home	☐ Keshav Nursing Home	☐ Escort Hospital
☐ Ballabgarh Nursing Home	☐ Manju Shree Nursing Home	☐ AIIMS - Delhi
☐ Kesar Nursing Home	☐ Sirohi Medical Center	☐ Other (specify):

2. If "Other", type of hospital: Public ☐ Private ☐ NGO ☐ Don't know ☐

3. HDSS ID of hospitalized person: vLirky esa Hkrhz O;fä dh HDSS igpku [X]-[X]-[X][X]-[X][X][X][X]-[ ][ ]-[ ][ ]

4. First name uke dk igyk fgLlk: ____

5. Last name uke dk vfUre fgLlk: ____

6. Date of Birth ___ ___ / ___ ___ / ___ ___ ___ ___ (dd/mm/yyyy)

7. Age mez: Years ___ ___ ___ Months ___ ___ (1-12)

8. Sex fyax: Male ☐ Female ☐

9. Illness related to hospitalization (please indicate all that apply):
vLirky esa Hkrhz gksus ls lacfU/kr chekjh ¼dksbZ ;k mu lc dk mYys[k djsa] tks ykxw gksrs gksa½

	Reason dkj.k	Yes gka	No ugha	Not Sure/Not Applicable iDdk ugha ykxw ugha
9a.	Diarrhea दस्त	☐	☐	☐
9b.	Fever बुखार	☐	☐	☐
9c.	Difficulty breathing/shortness of breath सांस लेने में कठिनाई/सांस फूलना	☐	☐	☐
9d.	Pneumonia or respiratory infection निमोनिया या सांस का संक्रमण	☐	☐	☐
9e.	Heart conditions हृदय रोग	☐	☐	☐
9f.	TB यक्ष्मा (टीबी)	☐	☐	☐
9g.	Chronic Lung Disease चिरकालिक फेफड़े का रोग	☐	☐	☐
9h.	Chronic Kidney Disease चिरकालिक गुर्दे का रोग	☐	☐	☐
9i.	Chronic Liver Disease चिरकालिक यकृत (लिवर) रोग	☐	☐	☐
9j.	Diabetes मधुमेह /शुगर	☐	☐	☐
9k.	HIV/AIDS एचआईवी/एड्स	☐	☐	☐
9l.	Seizures मिर्गी/दौरे पड़ना	☐	☐	☐
9m.	Neurological condition (specify): तंत्रिका संबंधी रोग (उल्लेख करें)	☐	☐	☐
9n.	Infants: Poor feeding/lethargy शिशु: कम खाना/सुस्त रहना	☐	☐	☐
9o.	Infantile paralysis शिशु पक्षाघात	☐	☐	☐
9p.	Other (specify): अन्य (उल्लेख करें)	☐	☐	☐
9q.	Other (specify): अन्य (उल्लेख करें)	☐	☐	☐
9r.	Comment:			

FAMILY: ___/____(New family number / Total number of new families) HOUSEHOLD HDSS ID: [1] [ ] [ ][ ] [ ][ ][ ][ ]

PROJECT PHC No. VILLAGE No. HOUSE No.

Burden of Severe Influenza in Rural India

10. Has this person ever been told by a healthcare provider that they have any of the following illnesses? (Check all that apply) D;k fdlh LokLF; dehZ us bl 0;fä ds ckjs esa dHkh ;g dgk fd mls fuEufyf[kRk esa ls dksbZ chekjh gS\ ¼d i ;k mu lc dk mYys[k djsa tks ykxw gksrs gksa½

☐ Diabetes मधुमेह /शुगर	☐ Chronic liver disease चिरकालिक यकृत(लिवर) रोग
☐ Asthma दमा	☐ HIV/AIDS एचआईवी/एड्स
☐ Chronic lung disease चिरकालिक फेफड़े का रोग	☐ Tuberculosis टी.बी.
☐ Heart condition हृदय रोग	☐ Neurological condition तंत्रिका संबंधी बीमारी,
☐ Stroke/CVA आघात/सीवीए	☐ Chronic renal disease चिरकालिक गुर्दे का रोग
☐ Seizures दैरे	☐ Other (specify): अन्य (उल्लेख करें)

11. How many days was this person ill before going to the hospital? vLirky esa tkus ls igys ;g 0;fä fdrus fnuksa ls chekj py jgk Fkk\ ___ ___ ___ days

12. Length of hospitalization in nights: fdruh jkr vLirky esa #duk iMk \ ___ ___ ___ nights

13. Was other medical care sought before hospitalization? D;k vLirky esa HkrhZ gksus ls igys vU; fpfdRlk yh xbZ Fkh\ Yes gka ☐ No ugha ☐ Don't Know iDdk ugha ☐

IF YES, COMPLETE QUESTION 14. ;fn gka] rks iz'u 14 HkjsA

IF NO OR DON'T KNOW, SKIP TO QUESTION 15. ;fn ugha ;k ekyqe ugha] rks iz'u 15 HkjsA

14. When and where was medical care sought for this problem before this hospitalization.
PLEASE LIST UP TO THREE LOCATIONS WHERE MEDICAL CARE WAS SOUGHT, STARTING WITH THE MOST RECENT. vLirky esa HkrhZ gksus ls igys bl jksx dk fy, fpfdRlk lqfo/kk dc vkSj dgka ls yh xbZ Fkh\ vkf[kjh mipkj ls ihNs pyRs gq, gj ml Tkxg ¼3 Tkxgksa Rkd½ ds ckjs esa cRkk;s Tkgka ls bykTk djok;k x;k FkkA

**Number of days before hospitalization:** HkrhZ gksus ls fdrus fnu igys fn[kk;k \	**Type of care sought:** fdl izdkj dk mipkj fy;k x;k\
**a**<br>___ ___ ___<br>**Nasal/throat swab taken?** D;k ukd@xys ls Lokc fy;k x;k \ ☐	☐ Government Hospital, Clinic or Health Centre सरकारी अस्पताल, क्लिनिक या स्वास्थ्य केन्द्र<br>☐ Private General Practitioner (MBBS, BAMS, BHMS) प्राइवेट चिकित्सक (एमबीबीएस/बीएएमएस/ बीएचएमएस डिगरी)<br>☐ Unqualified practitioner, traditional healer or RMP अयोग्य (झोलाछाप) चिकित्सक, पारंपरिक उपचारकर्ता या आर0एम0पी0<br>☐ Pharmacist/chemist फार्मेसिस्ट/ कैमिस्ट<br>☐ Religious leader धार्मिक गुरु<br>☐ Relative or friend संबंधी या दोस्त<br>☐ Other store or market अन्य स्टोर या बाजार

Burden of Severe Influenza in Rural India

**b**<br><br>___ ___ ___<br><br>**Nasal/throat swab taken?**<br>D;k ukd@xys ls Lokc<br>fy;k x;k \ ☐	☐ Government Hospital, Clinic or Health Centre सरकारी अस्पताल, क्लिनिक या स्वास्थ्य केन्द्र<br>☐ Private General Practitioner (MBBS, BAMS, BHMS) प्राइवेट चिकित्सक (एमबीबीएस/बीएएमएस/ बीएचएमएस डिगरी)<br>☐ Unqualified practitioner, traditional healer or RMP अयोग्य (झोलाछाप) चिकित्सक, पारंपरिक उपचारकर्ता या आर0एम0पी0<br>☐ Pharmacist/chemist फार्मेसिस्ट/ कैमिस्ट<br>☐ Religious leader धार्मिक गुरु<br>☐ Relative or friend संबंधी या दोस्त<br>☐ Other store or market अन्य स्टोर या बाजार
**c**<br><br>___ ___ ___<br><br>**Nasal/throat swab taken?**<br>D;k ukd@xys ls Lokc<br>fy;k x;k \ ☐	☐ Government Hospital, Clinic or Health Centre सरकारी अस्पताल, क्लिनिक या स्वास्थ्य केन्द्र<br>☐ Private General Practitioner (MBBS, BAMS, BHMS) प्राइवेट चिकित्सक (एमबीबीएस/बीएएमएस/ बीएचएमएस डिगरी)<br>☐ Unqualified practitioner, traditional healer or RMP अयोग्य (झोलाछाप) चिकित्सक, पारंपरिक उपचारकर्ता या आर0एम0पी0<br>☐ Pharmacist/chemist फार्मेसिस्ट/ कैमिस्ट<br>☐ Religious leader धार्मिक गुरु<br>☐ Relative or friend संबंधी या दोस्त<br>☐ Other store or market अन्य स्टोर या बाजार

15. What was the outcome of this hospitalization? bl vLirky esa HkrhZ gksus dk D;k ifj.kke Fkk\

- ☐ Sent home and still alive. घर भेजा गया और अब सही है।
- ☐ Still hospitalized अभी अस्पताल में भर्ती है।
- ☐ Died at hospital अस्पताल में मृत्यु हो गई।
- ☐ Died after release from hospital: Period after which death occurred: ___ ___ weeks ___ ___ months
  अस्पताल से छुट्टी के बाद &&& lIrkg] &&& ekg &&& वर्ष में मृत्यु हो गई।

END OF HOSPITALIZATION PROFORMA FOR THIS HOSPITALIZATION
KINDLY FILL SUPPLEMENTARY HOSPITALIZATION PROFORMA FOR ANY OTHER HOSPITALIZATION IN THE FAMILY

FAMILY: ___/____(New family number / Total number of new families) HOUSEHOLD HDSS ID: [1] PROJECT [ ] PHC No. [ ][ ] VILLAGE No. [ ][ ][ ][ ] HOUSE No.

Burden of Severe Influenza in Rural India

Proforma 3: Health Utilization Survey

Part 1-B: Hospitalization Proforma Hkkx 1&[k % vLirky izksQkekZ

COMPLETE THIS PROFORMA FOR EACH HOSPITALIZATION DUE TO "ACUTE MEDICAL ILLNESS" OR "OTHER" FROM HOSPITALIZATION TABLE IN PART 1-A

ÞxaHkhj fpfdRlh; chekjhß ;k ÞvU;ß dh dkj.k iR;sd ckj vLirky esa HkrhZ gksus dh lwpuk esa bl izksQkekZ dks Hkkx 1&d esa nh xbZ vLirky esa HkrhZ gksus dh rkfydk ls HkjsA

**1. Name of hospital where admitted:** vLirky dk uke		
☐ Ramesh Nursing Home	☐ Gupta Hospital	☐ Akash Hospital
☐ Manish Hospital	☐ Jindal Nursing Home	☐ Civil Hospital, Ballabgarh
☐ Arya Nursing Home	☐ Mittal Hospital	☐ BK Hospital
☐ Dr. Sudarshan Gupta Hospital	☐ Prakash Hospital	☐ Sun Flag Hospital
☐ Bhatia Nursing Home	☐ Keshav Nursing Home	☐ Escort Hospital
☐ Ballabgarh Nursing Home	☐ Manju Shree Nursing Home	☐ AIIMS - Delhi
☐ Kesar Nursing Home	☐ Sirohi Medical Center	☐ Other (specify):

**2. If "Other", type of hospital:**	Public ☐ Private ☐ NGO ☐ Don't know ☐
**3. HDSS ID of hospitalized person:** vLirky esa HkrhZ O;fä dh ,pMh,l,l igpku	[x]-[x]-[x][x]-[x][x][x][x]-[ ][ ]-[ ][ ]
**4. First name** uke dk igyk fgLlk:	
**5. Last name** uke dk vfUre fgLlk:	
**6. Date of Birth**	___ ___ / ___ ___ / ___ ___ ___ ___ (dd/mm/yyyy)
**7. Age** mez:	Years ___ ___ ___ Months ___ ___ (1-12)
**8. Sex** fyax:	Male ☐ Female ☐

9. Illness related to hospitalization (please indicate all that apply):
vLirky esa HkrhZ gksus ls lacaf/kr chekjh ¼d`i;k mu lc dk mYys[k djsa tks ykxw gksrs gksa½

	**Reason** dkj.k	**Yes** gka	**No** ugha	**Not Sure /** iDdk ugha
**9a.**	Diarrhea दस्त	☐	☐	☐
**9b.**	Fever बुखार	☐	☐	☐
**9c.**	Difficulty breathing/shortness of breath सांस लेने में कठिनाई/सांस फूलना	☐	☐	☐
**9d.**	Pneumonia or respiratory infection निमोनिया या सांस का संक्रमण	☐	☐	☐
**9e.**	Heart conditions हृदय रोग	☐	☐	☐
**9f.**	TB यक्ष्मा (टीबी)	☐	☐	☐
**9g.**	Chronic Lung Disease चिरकालिक फेफड़े का रोग	☐	☐	☐
**9h.**	Chronic Kidney Disease चिरकालिक गुर्दे का रोग	☐	☐	☐
**9i.**	Chronic Liver Disease चिरकालिक यकृत(लिवर) रोग	☐	☐	☐
**9j.**	Diabetes मधुमेह /शुगर	☐	☐	☐
**9k.**	HIV/AIDS एचआईवी/एड्स	☐	☐	☐
**9l.**	Seizures मिर्गी/दौरे पड़ना	☐	☐	☐
**9m.**	Neurological condition (specify): तंत्रिका संबंधी रोग (उल्लेख करें)	☐	☐	☐
**9n.**	Infants: Poor feeding/lethargy शिशु: कम खाना/सुस्त रहना	☐	☐	☐
**9o.**	Infantile paralysis शिशु पक्षाघात	☐	☐	☐

Burden of Severe Influenza in Rural India

**9p.**	Other (specify): अन्य (उल्लेख करें)	☐	☐	☐
**9q.**	Other (specify): अन्य (उल्लेख करें)	☐	☐	☐
**9r.**	Comment:			

10. Has this person ever been told by a healthcare provider that they have any of the following illnesses? (Check all that apply) D;k fdlh LokLF; deh u bl 0;fä dks crk;k gS fd bldks ;g chekjh fd ml s fuEufyf[kr esa ls dksbZ chekjh gS\ ¼dk;k mu lc dk mYys[k djsa tks ykxw gksrh gks½

☐ Diabetes मधुमेह /शुगर	☐ Chronic liver disease चिरकालिक यकृत(लिवर) रोग
☐ Asthma दमा	☐ HIV/AIDS एचआईवी/एड्स
☐ Chronic lung disease चिरकालिक फेफड़े का रोग	☐ Tuberculosis टी.बी.
☐ Heart condition हृदय रोग	☐ Neurological condition तंत्रिका संबंधी बीमारी,
☐ Stroke/CVA आघात/सीवीए	☐ Chronic renal disease चिरकालिक गुर्दे का रोग
☐ Seizures दौरे	☐ Other (specify): अन्य (उल्लेख करें)

**11. How many days was this person ill before going to the hospital?** vLirky esa tkus ls igys ;g 0;fä fdrus fnuksa ls chekj py jgk Fkk\	___ ___ ___ days
**12. Length of hospitalization in nights:** fdruh jkr vLirky esa #duk iMk \	___ ___ ___ nights
**13. Was other medical care sought before hospitalization?** D;k vLirky esa HkrhZ gksus ls igys vU; fpfdRlk yh xbZ Fkh\	Yes gka ☐ No ugha ☐ Don't Know iDdk ugha ☐

IF YES, COMPLETE QUESTION 14. ;fn gka] rks iz'u 14 HkjsA

IF NO OR DON'T KNOW, SKIP TO QUESTION 15. ;fn ugha ;k ekywe ugha] rks iz'u 15 HkjsA

14. When and where was medical care sought for this problem before this hospitalization.
PLEASE LIST UP TO THREE LOCATIONS WHERE MEDICAL CARE WAS SOUGHT, STARTING WITH THE MOST RECENT. vLirky esa HkrhZ gksus ls igys bl jksx dk fy, fpfdRlk lqfo/kk dc vkSj dgka ls yh xbZ Fkh\ vkf[kjh mipkj ls 'kq: djrs gq, gj ml txg ¼3 txgksa rd½ ds ckjs esa crk;s tgka ls bykt djok;k x;k FkkA

**Number of days before hospitalization:** HkrhZ gksus ls fdrus fnu igys fn[kk;k \	**Type of care sought:** fdl izdkj dk mipkj fy;k x;k\
**a** ___ ___ ___ **Nasal/throat swab taken?** D;k ukd@xys ls Lokc fy;k x;k \ ☐	☐ Government Hospital, Clinic or Health Centre सरकारी अस्पताल, क्लिनिक या स्वास्थ्य केन्द्र<br>☐ Private General Practitioner (MBBS, BAMS, BHMS) प्राइवेट चिकित्सक (एमबीबीएस/बीएएमएस/ बीएचएमएस डिगरी)<br>☐ Unqualified practitioner, traditional healer or RMP अयोग्य (झोलाछाप) चिकित्सक, पारंपरिक उपचारकर्ता या आर0एम0पी0<br>☐ Pharmacist/chemist फार्मेसिस्ट/ कैमिस्ट<br>☐ Religious leader धार्मिक गुरु<br>☐ Relative or friend संबंधी या दोस्त<br>☐ Other store or market अन्य स्टोर या बाजार

Burden of Severe Influenza in Rural India

**b**<br><br>___ ___ ___<br><br>**Nasal / throat swab taken?**<br>D;k ukd@xys ls Lokc fy;k x;k \ ☐	☐ Government Hospital, Clinic or Health Centre सरकारी अस्पताल, क्लिनिक या स्वास्थ्य केन्द्र<br>☐ Private General Practitioner (MBBS, BAMS, BHMS) प्राइवेट चिकित्सक (एमबीबीएस/बीएएमएस/ बीएचएमएस डिगरी)<br>☐ Unqualified practitioner, traditional healer or RMP अयोग्य (झोलाछाप) चिकित्सक, पारंपरिक उपचारकर्ता या आर0एम0पी0<br>☐ Pharmacist/chemist फार्मेसिस्ट/ कैमिस्ट<br>☐ Religious leader धार्मिक गुरु<br>☐ Relative or friend संबंधी या दोस्त<br>☐ Other store or market अन्य स्टोर या बाजार
**c**<br><br>___ ___ ___<br><br>**Nasal / throat swab taken?**<br>D;k ukd@xys ls Lokc fy;k x;k \ ☐	☐ Government Hospital, Clinic or Health Centre सरकारी अस्पताल, क्लिनिक या स्वास्थ्य केन्द्र<br>☐ Private General Practitioner (MBBS, BAMS, BHMS) प्राइवेट चिकित्सक (एमबीबीएस/बीएएमएस/ बीएचएमएस डिगरी)<br>☐ Unqualified practitioner, traditional healer or RMP अयोग्य (झोलाछाप) चिकित्सक, पारंपरिक उपचारकर्ता या आर0एम0पी0<br>☐ Pharmacist/chemist फार्मेसिस्ट/ कैमिस्ट<br>☐ Religious leader धार्मिक गुरु<br>☐ Relative or friend संबंधी या दोस्त<br>☐ Other store or market अन्य स्टोर या बाजार

15. What was the outcome of this hospitalization? bl vLirky esa HkrhZ gksus dk D;k ifj.kke Fkk\

- ☐ Sent home and still alive. घर भेजा गया और अब सही है।
- ☐ Still hospitalized अभी अस्पताल में भर्ती है।
- ☐ Died at hospital अस्पताल में मृत्यु हो गई।
- ☐ Died after release from hospital: Period after which death occurred: ___ ___ weeks ___ ___ months
  अस्पताल से छुट्टी के बाद &&& lIrkg] &&& ekg &&& वर्ष में मृत्यु हो गई।

END OF HOSPITALIZATION PROFORMA FOR THIS HOSPITALIZATION
KINDLY FILL SUPPLEMENTARY HOSPITALIZATION PROFORMA FOR ANY OTHER HOSPITALIZATION IN THE FAMILY

Burden of Severe Influenza in Rural India

Proforma 3: Health Utilization Survey

Part 1-B: Hospitalization Proforma Hkkx 1&[k % vLirky izksQkekZ

COMPLETE THIS PROFORMA FOR EACH HOSPITALIZATION DUE TO "ACUTE MEDICAL ILLNESS" OR "OTHER" FROM HOSPITALIZATION TABLE IN PART 1-A

ÞxEHkhj fpfdRlh; chekjhß ;k ÞvU;ß ds dkj.k iR;sd ckj vLirky esa HkrhZ gksus ds lEcU/k esa bl izksQkekZ dks Hkkx 1&d esa nh xbZ vLIrky esa HkrhZ gksus dh rkfydk ls HkjsA

**1. Name of hospital where admitted:** vLirky dk uke	
☐ Ramesh Nursing Home	☐ Gupta Hospital	☐ Akash Hospital
☐ Manish Hospital	☐ Jindal Nursing Home	☐ Civil Hospital, Ballabgarh
☐ Arya Nursing Home	☐ Mittal Hospital	☐ BK Hospital
☐ Dr. Sudarshan Gupta Hospital	☐ Prakash Hospital	☐ Sun Flag Hospital
☐ Bhatia Nursing Home	☐ Keshav Nursing Home	☐ Escort Hospital
☐ Ballabgarh Nursing Home	☐ Manju Shree Nursing Home	☐ AIIMS - Delhi
☐ Kesar Nursing Home	☐ Sirohi Medical Center	☐ Other (specify):

**2. If "Other", type of hospital:**	Public ☐ Private ☐ NGO ☐ Don't know ☐
**3. HDSS ID of hospitalized person:** vLirky esa HkrhZ O;fä dh ,pMh,l,l igpku	[x]-[x]-[x][x]-[x][x][x][x]-[ ][ ]-[ ][ ]
**4. First name** uke dk igyk fgLlk:	
**5. Last name** uke dk vfare fgLlk:	
**6. Date of Birth**	___ ___ / ___ ___ / ___ ___ ___ ___ (dd/mm/yyyy)
**7. Age** mez:	Years ___ ___ ___ Months ___ ___ (1-12)
**8. Sex** fyax:	Male ☐ Female ☐

9. Illness related to hospitalization (please indicate all that apply):
vLirky esa HkrhZ gksus ls lEcf/kr chekjh ¼dfi;k mu lc dk mYys[k djsa tks ykxw gksrs gks½

	Reason dkj.k	Yes gka	No ugha	Not Sure / iDdk ugha
9a.	Diarrhea दस्त	☐	☐	☐
9b.	Fever बुखार	☐	☐	☐
9c.	Difficulty breathing/shortness of breath सांस लेने में कठिनाई/सांस फूलना	☐	☐	☐
9d.	Pneumonia or respiratory infection निमोनिया या सांस का संक्रमण	☐	☐	☐
9e.	Heart conditions हृदय रोग	☐	☐	☐
9f.	TB यक्ष्मा (टीबी)	☐	☐	☐
9g.	Chronic Lung Disease चिरकालिक फेफड़े का रोग	☐	☐	☐
9h.	Chronic Kidney Disease चिरकालिक गुर्दे का रोग	☐	☐	☐
9i.	Chronic Liver Disease चिरकालिक यकृत(लिवर) रोग	☐	☐	☐
9j.	Diabetes मधुमेह /शुगर	☐	☐	☐
9k.	HIV/AIDS एचआईवी/एड्स	☐	☐	☐
9l.	Seizures मिर्गी/दौरे पड़ना	☐	☐	☐
9m.	Neurological condition (specify): तंत्रिका संबंधी रोग (उल्लेख करें)	☐	☐	☐
9n.	Infants: Poor feeding/lethargy शिशु: कम खाना/सुस्त रहना	☐	☐	☐
9o.	Infantile paralysis शिशु पक्षाघात	☐	☐	☐

FAMILY: ___/____(New family number / Total number of new families) HOUSEHOLD HDSS ID: [1] [ ] [ ][ ] [ ][ ][ ][ ]
PROJECT PHC No. VILLAGE No. HOUSE No.

Burden of Severe Influenza in Rural India

9p.	Other (specify): अन्य (उल्लेख करें)	☐	☐	☐
9q.	Other (specify): अन्य (उल्लेख करें)	☐	☐	☐
9r.	Comment:			

10. Has this person ever been told by a healthcare provider that they have any of the following illnesses? (Check all that apply) D;k fdlh LokLF; deh us bl 0;fä dks ckj esa dHh ;g dgk fd mls fuEufyf[kr esa ls dksbZ chekjh gS\ ¼dks;k mu lc dk mYys[k djsa] tks ykxw gksrs gksa½

☐ Diabetes मधुमेह /शुगर	☐ Chronic liver disease चिरकालिक यकृत(लिवर) रोग
☐ Asthma दमा	☐ HIV/AIDS एचआईवी/एड्स
☐ Chronic lung disease चिरकालिक फेफड़े का रोग	☐ Tuberculosis टी.बी.
☐ Heart condition हृदय रोग	☐ Neurological condition तंत्रिका संबंधी बीमारी,
☐ Stroke/CVA आघात/सीवीए	☐ Chronic renal disease चिरकालिक गुर्दे का रोग
☐ Seizures दौरे	☐ Other (specify): अन्य (उल्लेख करें)

**11. How many days was this person ill before going to the hospital?** vLirky esa tkus ls igys ;g 0;fä fdrus fnuksa ls chekj py jgk Fkk\	___ ___ ___ days
**12. Length of hospitalization in nights:** fdruh jkr vLirky esa #duk iM+k \	___ ___ ___ nights
**13. Was other medical care sought before hospitalization?** D;k vLirky esa HkrhZ gksus ls igys vU; fpfdRlk yh xbZ Fkh\	Yes gk ☐ No ugh ☐ Don't Know iDdk ugh ☐

IF YES, COMPLETE QUESTION 14. ;fn gk] rks iz'u 14 HkjsA

IF NO OR DON'T KNOW, SKIP TO QUESTION 15. ;fn ugh ;k ekyqe ugh] rks iz'u 15 HkjsA

14. When and where was medical care sought for this problem before this hospitalization.
PLEASE LIST UP TO THREE LOCATIONS WHERE MEDICAL CARE WAS SOUGHT, STARTING WITH THE MOST RECENT. vLirky esa HkrhZ gksus ls igys bl jksx ds fy, fpfdRlk lifo/kk dc vkSj dgk ls yh xbZ Fkh\ vkf[kjh mipkj ls 'kq: djrs gq, gj mlTkxg ¼3 Tkxgks rd½ ds ckjs esa crk;s Tkgka ls bykTk djok;k x;k FkkA

Number of days before hospitalization: HkrhZ gksus ls fdrus fnu igys fn[kk;k \	Type of care sought: fdl izdkj dk mipkj fy;k x;k\
**a** ___ ___ ___ **Nasal/throat swab taken?** D;k ukd@xys ls Lokc fy;k x;k \ ☐	☐ Government Hospital, Clinic or Health Centre सरकारी अस्पताल, क्लिनिक या स्वास्थ्य केन्द्र ☐ Private General Practitioner (MBBS, BAMS, BHMS) प्राइवेट चिकित्सक (एमबीबीएस/बीएएमएस/ बीएचएमएस डिगरी) ☐ Unqualified practitioner, traditional healer or RMP अयोग्य (झोलाछाप) चिकित्सक, पारंपरिक उपचारकर्ता या आर०एम०पी० ☐ Pharmacist/chemist फार्मेसिस्ट/ कैमिस्ट ☐ Religious leader धार्मिक गुरु ☐ Relative or friend संबंधी या दोस्त ☐ Other store or market अन्य स्टोर या बाजार

Burden of Severe Influenza in Rural India

**b**<br><br>___ ___ ___<br><br>**Nasal/throat swab taken?**<br>D;k ukd@xys ls Lokc fy;k x;k \ ☐	☐ Government Hospital, Clinic or Health Centre सरकारी अस्पताल, क्लिनिक या स्वास्थ्य केन्द्र<br>☐ Private General Practitioner (MBBS, BAMS, BHMS) प्राइवेट चिकित्सक (एमबीबीएस/बीएएमएस/ बीएचएमएस डिगरी)<br>☐ Unqualified practitioner, traditional healer or RMP अयोग्य (झोलाछाप) चिकित्सक, पारंपरिक उपचारकर्ता या आर0एम0पी0<br>☐ Pharmacist/chemist फार्मेसिस्ट/ कैमिस्ट<br>☐ Religious leader धार्मिक गुरु<br>☐ Relative or friend संबंधी या दोस्त<br>☐ Other store or market अन्य स्टोर या बाजार
**c**<br><br>___ ___ ___<br><br>**Nasal/throat swab taken?**<br>D;k ukd@xys ls Lokc fy;k x;k \ ☐	☐ Government Hospital, Clinic or Health Centre सरकारी अस्पताल, क्लिनिक या स्वास्थ्य केन्द्र<br>☐ Private General Practitioner (MBBS, BAMS, BHMS) प्राइवेट चिकित्सक (एमबीबीएस/बीएएमएस/ बीएचएमएस डिगरी)<br>☐ Unqualified practitioner, traditional healer or RMP अयोग्य (झोलाछाप) चिकित्सक, पारंपरिक उपचारकर्ता या आर0एम0पी0<br>☐ Pharmacist/chemist फार्मेसिस्ट/ कैमिस्ट<br>☐ Religious leader धार्मिक गुरु<br>☐ Relative or friend संबंधी या दोस्त<br>☐ Other store or market अन्य स्टोर या बाजार

15. What was the outcome of this hospitalization? bl vLirky esa Hkrh gksus dk D;k ifj.kke Fkk\

- ☐ Sent home and still alive. घर भेजा गया और अब सही है।
- ☐ Still hospitalized अभी अस्पताल में भर्ती है।
- ☐ Died at hospital अस्पताल में मृत्यु हो गई।
- ☐ Died after release from hospital: Period after which death occurred: ___ ___ weeks ___ ___ months
  अस्पताल से छुट्टी के बाद &&& lIrkg] &&& ekg &&& वर्ष में मृत्यु हो गई।

END OF HOSPITALIZATION PROFORMA FOR THIS HOSPITALIZATION
KINDLY FILL SUPPLEMENTARY HOSPITALIZATION PROFORMA FOR ANY OTHER HOSPITALIZATION IN THE FAMILY

Proforma 3: Health Utilization Survey

Part 1-c: Recent Deaths Hkkx 1&x % gky esa gqbZ eR;q

**1.** During the last 12 months have any members of this household died? क्या पिछले बारह मास मे इस घर के किसी सदस्य की मृत्यु हुई है?	Yes gka ☐	No ugha ☐	Don't Know iDdk ugha ☐

IF YES, COMPLETE PROFORMA 4 ;fn gka] rks izQkekZ 4 Hkjsa
IF NO, END OF PROFORMA ;fn ugha] rks ;g izQkekZ lekIr
IF DON'T KNOW, MAKE ARRANGEMENTS TO RETURN WHEN APPROPRIATE INFORMANT IS AVAILABLE.

;fn ekywe ugha] rks nqckjk fQj fdlh fnu Äj ij vkus dk izcU/k djsa

Influenza Disease Burden in India – OPD Burden (NI-1100)

FORM ID: | B | 2 | | | | | |

CRHSP, Ballabgarh, Faridabad
All India Institute of Medical Sciences, New Delhi

Name of interviewer		Date		Time	
SITE:					

PART 1 - ELIGIBILITY SCREENING

1. **1.1** Residence: What village does this person live in?

☐ Ahmedpur (2-64)	☐ Fatehpur Billoch (1-40)	☐ Naryala (2-60)
☐ Atali (2-51)	☐ Garkhera (2-50)	☐ Nawada (1-31)
☐ Behbalpur (1-14)	☐ Jaya (2-61)	☐ Nirhawali (2-63)
☐ Bhatpura (1-43)	☐ Junehra (1-13)	☐ Panehra Kalan (2-62)
☐ Bukharpur (1-20)	☐ Khera (1-12)	☐ Phapunda (1-15)
☐ Chandawali (1-30)	☐ Ladholi (1-41)	☐ Shahpur Kalan (1-42)
☐ Chhainsa (2-70)	☐ Machgar (1-21)	☐ Sahupura (1-34)
☐ Dayalpur (1-11)	☐ Malerna (1-33)	☐ Sotai (1-22)
☐ Dayalpur Colony (1-10)	☐ Maujpur (2-52)	☐ Other (specify):
☐ Duleypur (2-71)	☐ Mujedhi (1-32)	
**1.2 Is the Patient a resident of DSS villages listed above?**		**YES** ☐ **NO** ☐

2. 2.1 Reason for OPD visit – Inclusion Criteria

2.1.a	Does this person have onset of new respiratory symptom(s) within the past 3 days?	YES ☐ NO ☐
2.1.b	Does this person have any new onset of medical illness in the past 3 days?	YES ☐ NO ☐
2.1.c	Is patient a child less than 1 year old who is presenting with fever as the only symptom with onset within last three days?	YES ☐ NO ☐
**2.2**	**If ANY of 2.1.a or 2.1.b or 2.1.c are marked as YES**	YES ☐ NO ☐

3. 3.1 Reason for OPD visit – Exclusion Criteria

3.1.a.	Did this visit result from trauma?	YES ☐ NO ☐
3.1.b.	Did this visit result from purely obstetrical needs?	YES ☐ NO ☐
3.1.c.	Did this visit result from accidental poisoning	YES ☐ NO ☐
3.1.d.	Did this visit result for getting an elective surgery or as a follow up to elective surgery?	YES ☐ NO ☐
3.1.e.	Did this visit result for diarrhea with no respiratory symptoms?	YES ☐ NO ☐
**3.2**	**If ANY of responses of 3.1.a - 3.1.e are YES , mark YES here.**	YES ☐ **NO** ☐

4. If *1.2 is YES*, *2.2 is YES* and *3.2 is NO:* then mark Yes here, else mark No: YES ☐ NO ☐

If 4 is NO, Complete 6 and 7 and Stop . If 4 is YES, Continue to Q 5

CONSENT

5. Inform the patient about study and take Consent. Does the patient give consent? YES ☐ NO ☐

If 5 is NO, Complete 6 and 7 and then Stop. If 5 is YES, Continue to main proforma

6. Age: ________________ yr ______________ months

7. Sex: MALE ☐ FEMALE ☐

 Ver. Date: Dec 7, 2010

Influenza Disease Burden in India – OPD Burden (NI-1100)

FORM ID: | B | 2 | | | | | |

अखिल भारतीय आयुर्विज्ञान संस्थान

सूचना सहमति पत्र

अध्ध्यन का शीर्षकः *"भारत के ग्रामीण क्षेत्रों में एनफ्लूऐन्जा वायरस का भार"*

अन्वेशकः **डॉ० एस० बरूर, एम्स, फोन न० 011-26594926**
डॉ० के० आनन्द, सिविल अस्पताल बल्लभगढ, एम्स, फोन न० 0129 2241362
डॉ० संजय कुमार राय, सिविल अस्पताल बल्लभगढ, एम्स, फोन न० 0129 2241362

सूचना पत्र में दी गई बातें मेरे द्वारा ध्यान पूर्वक पढ लिए गए हैं /मेरे द्वारा समझी जाने वाली भाषा में विस्तार से वर्णन किया गया है। मैने इन बातों को पूर्ण रूप से समझ लिया है।

शोध की प्रकृति व उद्वेश्य और संभावित जोखिम, लाभ और अनुमानित अवधि तथा अन्य सम्बंधित जानकारी का मेरे लिए विस्तार से वर्णन किया गया है। मैं यह जानता/जानती हूँ कि मेरा इस शोध में भाग लेना स्वैच्छिक है और मैं बिना कोई कारण बताए व चिकित्सकीय देखभाल तथा अपने वैधानिक अधिकारो को खोए बिना किसी भी समय इस शोध से स्वेच्छा से बाहर हो सकता/सकती हूँ।मैं यह जानता/जानती हूँ कि इस शोध में भाग लेने पर मेरे बारे में एकत्रित की गई जानकारी और मेरी चिकित्सा से संबंधित कोई भी अंश एम्स या इससे संबंधित किसी विशेष जिम्मेवार तथा नियंत्रक प्राधिकारियों द्वारा रखी जाएगी।मैं इन जिम्मेवार प्राधिकारियों को अपने रिकोर्ड को देखने की अनुमति देता/देती हूँ।

1. मै इस शोध में भाग लेने के लिए सहमत हूँ।

2. ☐ मैं अपने संम्पल को भविष्य में संक्रमण शोध के लिए सुरक्षित रखने की अनुमति देता/देती हूँ।
☐ मैं अपने संम्पल को भविष्य में संक्रमण शोध के लिए सुरक्षित रखने की **अनुमति नहीं देता/देती** हूँ।
☐ यदि मेरा सैम्पल भविष्य में संक्रमण शोध के लिए प्रयोग हो तो मुझे अधिसूचित किया जाए।

____
(हस्ताक्षर/बाए हाथ के अगूठे का निशान)

तिथिः ________________
स्थानः ________________

प्रतिभागी का नामः ______________________
पुत्र/पुत्री/पति/पत्निः ______________________
पूरा पताः____

यह प्रमाणित किया जाता है कि उपरोक्त मंजूरी मेरी मौजूदगी में ली गई है।

1.) साक्षी – **1**

____
हस्ताक्षर

नामः ____________
पताः ________________________________

2.) साक्षी – **2**

____
हस्ताक्षर

नामः ____________
पताः ________________________________

Influenza Disease Burden in India – OPD Burden (NI-1100)

FORM ID: | B | 2 | | | | | |

PART B – Out-Patient Profroma

8. CR Number	9. Name of Patient	10. Fathers Name
11. Date of Birth __/__/__ Not Known	12. Age (if DOB not known) ____ Years _____ Months	13. Contact number
14. Complete Address	15. Respondent ☐ Self ☐ Son/Daughter ☐ Parents ☐ Other (specify)________________	

CLINICAL HISTORY

**16. Chief Complaints**				
**17.**	**PLEASE CHECK YES, NO, OR NOT SURE FOR EACH SYMPTOM**	**Yes**	**No**	**Not Sure**
a.	Fever	☐	☐	☐
**IF NO FEVER, SKIP To Q 17.b**				
I.	Date of Onset of Fever (dd – mm - yyyy): ___ - ___ - ________			
II.	Chills or rigors	☐	☐	☐
b.	Rash	☐	☐	☐
c.	Cough	☐	☐	☐
**IF NO COUGH SKIP TO Q 17.d**				
I.	Productive cough?	☐	☐	☐
II.	Blood in sputum?	☐	☐	☐
III.	☐ New cough<br>☐ Worsening of existing cough			
d.	Any difficulty breathing / shortness of breath	☐	☐	☐
**IF NO DIFFIDULTY BREATHING SKIP TO Q 17.e**				
I.	☐ New difficulty breathing / shortness of breath<br>☐ Worsening of existing difficulty breathing / shortness of breath			
e.	Fast breathing (for Under 5 children)	☐	☐	☐
f.	Nasal discharge	☐	☐	☐
g.	Nasal stuffiness	☐	☐	☐
h.	Sore throat	☐	☐	☐
i.	Ear pain and/or ear discharge	☐	☐	☐
j.	Chest pain while breathing	☐	☐	☐
**IF ANY OF 17c – 17j are YES, GO TO 17k ELSE GO TO 17l**				
k.	Date of Onset of respiratory complaints (dd – mm – yyyy) _____ - _____ - ___________			
l.	Vomiting	☐	☐	☐
m.	Diarrhea	☐	☐	☐
n.	Jaundice (yellowish discoloration of eyes, palms, and soles)	☐	☐	☐
o.	Pain Abdomen	☐	☐	☐
p.	Inability/refusal to feed / decreased feeding (for under 5 children)	☐	☐	☐
q.	Lethargy (for Under 5 children)	☐	☐	☐
r.	Muscle aches / body ache	☐	☐	☐
s.	Headache	☐	☐	☐
t.	Seizure / convulsions	☐	☐	☐
u.	Confusion	☐	☐	☐
v.	Loss of consciousness	☐	☐	☐
w.	Comments / Other Relevant Symptoms:			

Influenza Disease Burden in India – OPD Burden (NI-1100)

FORM ID: | B | 2 | | | | | |

PRE-EXISTING CONDITIONS

**18. Have you ever been told by a healthcare provider that you have any of the following problems?**					**18.2 If YES, mark if the current visit related to a worsening the condition**
Check "Yes", "No" Or "Don't Know" For Each Condition		**Yes (go to 18.2)**	**No**	**Don't know**	
a.	Chronic lung disease including emphysema, COPD or chronic bronchitis	☐	☐	☐	☐
b.	Asthma	☐	☐	☐	☐
c.	Tuberculosis	☐	☐	☐	☐
d.	Heart condition (e.g., CAD, CHF, congenital or rheumatic heart disease)	☐	☐	☐	☐
e.	History of heart attack	☐	☐	☐	☐
f.	Hypertension or high blood pressure	☐	☐	☐	☐
g.	Stroke or CVA	☐	☐	☐	☐
h.	Chronic diarrhea in children under 5	☐	☐	☐	☐
i.	Chronic seizure disorder and/or history of seizures	☐	☐	☐	☐
j.	Neurological condition present since birth or early infancy	☐	☐	☐	☐
k.	Diabetes – Insulin dependent (IDDM)	☐	☐	☐	☐
l.	Diabetes – Non-insulin dependent (NIDDM)	☐	☐	☐	☐
m.	Chronic liver disease	☐	☐	☐	☐
n.	Thalassemia or other chronic anemia	☐	☐	☐	☐
o.	Chronic renal disease	☐	☐	☐	☐
p.	Immune deficiency or ongoing chemotherapy treatment/radiation therapy	☐	☐	☐	☐
q.	Malignancy	☐	☐	☐	☐
r.	Other (specify):	☐	☐	☐	☐

19. Are you currently pregnant?	Yes ☐	No ☐	Not applicable (Mark for males) ☐<br>Don't know ☐
20. Are you currently a smoker?	Yes ☐	No ☐	
21. Do any household members regularly smoke inside your home?	Yes ☐	No ☐	Don't know ☐

Influenza Disease Burden in India – OPD Burden (NI-1100)

FORM ID: | B | 2 | | | | | |

EXAMINATION

GENERAL PHYSICAL EXAMINATION

22.	Weight (for under 5)	_________________ Kg	☐ Not Taken
23.	Height (for under 5)	_________________ Cms	☐ Not Taken
24.	Mid Arm Circumference (for under 5)	_________________ Cms	☐ Not Taken
25.	Temperature	___________ ☐°F / ☐°C	☐ Not Taken
26.	Heart rate	__________ Per Minute	☐ Not Taken
27.	Respiratory rate	__________ Per Minute	☐ Not Taken
28.	Oxygen Saturation	________________ %	☐ Room Air<br>☐ Oxygen Therapy

	**PHYSICAL SIGNS**	**Yes**	**No**	**Not Sure**
29.	Pallor	☐	☐	☐
30.	Ear discharge	☐	☐	☐
31.	Nasal discharge	☐	☐	☐
32.	Sinus tenderness	☐	☐	☐
33.	Lymphadenopathy	☐	☐	☐
34.	Cyanosis	☐	☐	☐
35.	Clubbing	☐	☐	☐

RESPIRATORY SYSTEM EXAMINATION

	**Symptoms**	**Yes**	**No**	**Not Sure**
36.	Stridor	☐	☐	☐
37.	Lower chest in-drawing	☐	☐	☐
38.	Reduced air entry	☐	☐	☐
39.	Crepitations	☐	☐	☐
40.	Rhonchi / Wheezing	☐	☐	☐
41.	Bronchial Breathing	☐	☐	☐

42. OTHER SIGNIFICANT FINDINGS ON PHYSICAL EXAMINATION

SPECIMEN DETAILS

43.	Sample Collected	☐ YES	☐ NO
**IF NO, THEN SKIP Q 50 AND Q 51 AND COMPLETE PROFROMA**			
44.	Type of Sample Collected	a) ☐ Nasal Swab<br>b) ☐ Throat swab<br>c) ☐ Naso-pharyngeal aspirate<br>☐ Other (Specify)________________	
45.	Date and Time of Collection:	_____ / ______ / __________ ___ : ___ ☐AM / ☐PM	

48. Form Completed by: ☐ DOCTOR ☐ NURSE

Signature of Research Officer / Nurse
